# Defining albumin as a glycoprotein with multiple N-linked glycosylation sites

**DOI:** 10.1186/s12967-024-05000-5

**Published:** 2024-05-13

**Authors:** Kishore Garapati, Anu Jain, Benjamin J. Madden, Dong-Gi Mun, Jyoti Sharma, Rohit Budhraja, Akhilesh Pandey

**Affiliations:** 1 Manipal Academy of Higher Education (MAHE), Manipal, Karnataka India; 2 Institute of Bioinformatics, International Technology Park, Bangalore, Karnataka India; 3 Department of Laboratory Medicine and Pathology, Mayo Clinic, 200 First Street SW, Rochester, MN 55905 USA; 4 Proteomics Core, Mayo Clinic, Rochester, MN USA; 5 Center for Individualized Medicine, Mayo Clinic, Rochester, MN USA

**Keywords:** Human serum albumin, HSA, BSA, Glycoproteomics, Microheterogeneity, Novel glycosylation site

## Abstract

**Background:**

Glycosylation is an enzyme-catalyzed post-translational modification that is distinct from glycation and is present on a majority of plasma proteins. *N*-glycosylation occurs on asparagine residues predominantly within canonical *N*-glycosylation motifs (Asn-X-Ser/Thr) although non-canonical *N*-glycosylation motifs Asn-X-Cys/Val have also been reported. Albumin is the most abundant protein in plasma whose glycation is well-studied in diabetes mellitus. However, albumin has long been considered a non-glycosylated protein due to absence of canonical motifs. Albumin contains two non-canonical *N*-glycosylation motifs, of which one was recently reported to be glycosylated.

**Methods:**

We enriched abundant serum proteins to investigate their N-linked glycosylation followed by trypsin digestion and glycopeptide enrichment by size-exclusion or mixed-mode anion-exchange chromatography. Glycosylation at canonical as well as non-canonical sites was evaluated by liquid chromatography–tandem mass spectrometry (LC–MS/MS) of enriched glycopeptides. Deglycosylation analysis was performed to confirm N-linked glycosylation at non-canonical sites. Albumin-derived glycopeptides were fragmented by MS3 to confirm attached glycans. Parallel reaction monitoring was carried out on twenty additional samples to validate these findings. Bovine and rabbit albumin-derived glycopeptides were similarly analyzed by LC–MS/MS.

**Results:**

Human albumin is *N*-glycosylated at two non-canonical sites, Asn^68^ and Asn^123^. *N*-glycopeptides were detected at both sites bearing four complex sialylated glycans and validated by MS3-based fragmentation and deglycosylation studies. Targeted mass spectrometry confirmed glycosylation in twenty additional donor samples. Finally, the highly conserved Asn^123^ in bovine and rabbit serum albumin was also found to be glycosylated.

**Conclusions:**

Albumin is a glycoprotein with conserved N-linked glycosylation sites that could have potential clinical applications.

**Supplementary Information:**

The online version contains supplementary material available at 10.1186/s12967-024-05000-5.

## Background

Glycosylation is the commonest post-translational modification (PTM) of proteins [1]. It is distinct from glycation, a non-enzymatic process of protein modification by the addition of sugars on a background of hyperglycemia. Glycation affects a number of plasma proteins including albumin, haptoglobin and fibrinogen and is associated with microvascular damage and organ dysfunction in advanced diabetes [2]. By contrast, glycosylation is an enzyme-catalyzed physiological process which occurs on specific amino acids and is essential for protein stability, folding and function [3]. N-linked glycosylation is the most complex form of protein glycosylation in humans, where oligosaccharide chains or glycans are covalently attached to proteins at asparagine (Asn) residues by an *N*-glycosidic bond [1]. Most secretory and plasma proteins are *N*-glycosylated at asparagines in a canonical motif in the primary amino acid sequence, Asn-X-Ser/Thr, where X is any amino acid except proline [4]. The hydroxyl group in the side chain of serine or threonine performs the hydrogen bond donor function that is necessary for the catalytic transfer of the *N*-glycan to asparagine [5]. However, the presence of this motif is not sufficient for, and does not always result in, glycosylation. It is estimated that only ~ 70% of such sites are glycosylated [4]. Further, *N*-glycosylation sites are occupied by glycans to different levels, defining glycosylation macroheterogeneity [6]. Besides the canonical motif, *N*-glycosylation occurs on asparagines within the non-canonical motif Asn-X-Cys of some proteins, with the sulfhydryl group of cysteine performing the hydrogen bond donor function. However, the sulfur on cysteine has less electronegativity than oxygen on the side chains of serine or threonine [7]. As a result, this motif is known to be glycosylated at low levels in several proteins including transferrin and von Willebrand Factor [8, 9]. Another non-canonical motif, Asn-X-Val, has been shown to be glycosylated to low levels in some proteins including alpha-1B-glycoprotein and apolipoprotein B-100 [10, 11].

Mass spectrometry (MS)-based analysis of deglycosylated peptides has historically played an important role in the identification of glycoproteins and their sites of *N*-glycosylation [12]. Advancements in MS technology over the past several years coupled with the development of appropriate database search tools have facilitated comprehensive glycopeptide profiling with identification of intact glycans and their sites of attachment [11]. We sought to deploy advanced MS methods to discover and characterize glycosylation events that might have been missed previously because of low abundance or because they occurred at non-canonical motifs. Among abundant plasma proteins, such motifs, i.e., Asn-X-Cys or Asn-X-Val are present in alpha-2-macroglobulin, alpha-1-acid glycoprotein 2, transferrin, immunoglobulin heavy chains, and albumin [13]. Albumin is the most abundant plasma protein and besides maintenance of colloidal osmotic pressure of plasma, it functions as a transporter, antioxidant and enzyme [14]. It has been considered a non-glycosylated protein because it does not contain a canonical motif in its amino acid sequence. However, asparagines at sites Asn^68^ and Asn^123^ are part of non-canonical *N*-glycosylation motifs Asn-X(Glu)-Val and Asn-X(Glu)-Cys, respectively [13]. We wondered if albumin is glycosylated at these sites at levels that might not be detected by traditional methods of glycoprotein analysis [15]. Recently, one of these sites, i.e., Asn^68^, was reported to be linked to two glycans (Hex_5_HexNAc_4_NeuAc_2_ and Hex_5_HexNAc_4_NeuAc_1_) based on MS/MS fragmentation data [10]. In our experience with the analysis of plasma and serum-derived glycopeptides enriched using alternate methods, we observe a greater degree of glycan microheterogeneity in glycopeptides derived from abundant plasma proteins [11]. We were intrigued if Asn^68^ is occupied by a larger glycan repertoire and if Asn^123^ is also glycosylated. Thus, we systematically investigated N-linked glycosylation of albumin in serum from volunteer donors using a multi-pronged approach.

## Methods

### Samples

Twenty-three serum samples used in this study were deidentified residual samples from volunteer donors (approved by Mayo Clinic IRB: 21-012890).

### LC–MS/MS-based discovery analysis of serum-derived glycopeptides

Serum samples from volunteer donors were first enriched for 14 abundant serum proteins and digested with trypsin. Glycopeptides were enriched from the peptide mixture using either size exclusion chromatography or mixed-mode anion exchange cartridge (MAX), and analyzed by mass spectrometry (MS) in data dependent acquisition mode an Orbitrap Eclipse mass spectrometer (Thermo Fisher Scientific) [11, 16, 17]. Data was searched in pGlyco3 [18]. Commercial bovine (Thermo Scientific) and rabbit (Sigma) serum albumin were digested followed by glycopeptide enrichment using MAX. Details of sample preparation and MS analysis are provided in Additional file 1: Supplemental Methods.

### Mapping N-glycosylation sites onto structure of albumin

The crystal structure of human albumin derived from pooled human plasma with the identifier 1AO6 [19] was obtained from the PDB [20] and visualized using PyMOL (v2.5.7) [21]. N-linked glycosylation site Asn^68^ was highlighted in red color. The structure was rotated by 90º to visualize the other glycosylation site, Asn^123^, which was also highlighted in red.

### Deglycosylation analysis of serum glycoproteins

Glycopeptides from serum proteins enriched by MAX were treated overnight with PNGase F (N-Zyme Scientifics) in either ^16^O or ^18^O water (97% ^18^O enriched, Sigma) at 37 °C. Deglycosylated peptides were analyzed by MS in parallel reaction monitoring mode as described in the Additional file 1: Supplemental Methods. Spectral inspection and peak identification were done manually.

### MS3 analysis of glycopeptides

Albumin was immunoprecipitated from pooled serum samples using anti-albumin antibody (Invitrogen) followed by trypsin digestion and MAX-enrichment of glycopeptides. Selected glycopeptides were analyzed in the MS3 mode on an Orbitrap Eclipse mass spectrometer. Precursor ions were detected in the Orbitrap at a resolution of 120,000 with a scan range of 800 to 1500 m/z. Precursor ions were selected and fragmented in the ion-trap using collision induced dissociation (CID). Fragment ions were detected in the ion-trap and selected fragment ions for each precursor were further fragmented using HCD. Data analysis and fragment annotation in MS2 and MS3 spectra was done manually. See Additional file 1: Supplemental Methods for details.

### Targeted LC–MS/MS analysis

Glycopeptides derived from 20 volunteer donor serum samples were analyzed in targeted mode on an Orbitrap Exploris 480 mass spectrometer (Thermo Fisher Scientific) coupled with Ultimate 3000 liquid chromatography system. Inclusion list consisted of precursor ions for all the detected albumin glycopeptides. Data was analyzed using Skyline (v 22.2) [22]. Details are described in the Additional file 1: Supplemental Methods.

## Results

We employed a rigorous multi-step LC–MS/MS approach to detect and confirm *N*-glycosylation at the two non-canonical sites of albumin along with attached glycans. First, we performed deep discovery analysis using donor serum samples to identify intact glycopeptides with sites Asn^68^ and Asn^123^. We then confirmed our findings using streamlined enrichment methods, targeted LC–MS/MS analysis of ^18^O-labeled deglycosylated peptides as well as MS3 analysis of intact glycopeptides. These findings were validated in serum samples from twenty additional donors by targeted glycopeptide detection. Further, we show that the highly conserved glycosylation motif at Asn^123^ is also glycosylated in bovine and rabbit serum albumin.

### A novel N-linked glycosylation site on albumin

**Fig. 1:**
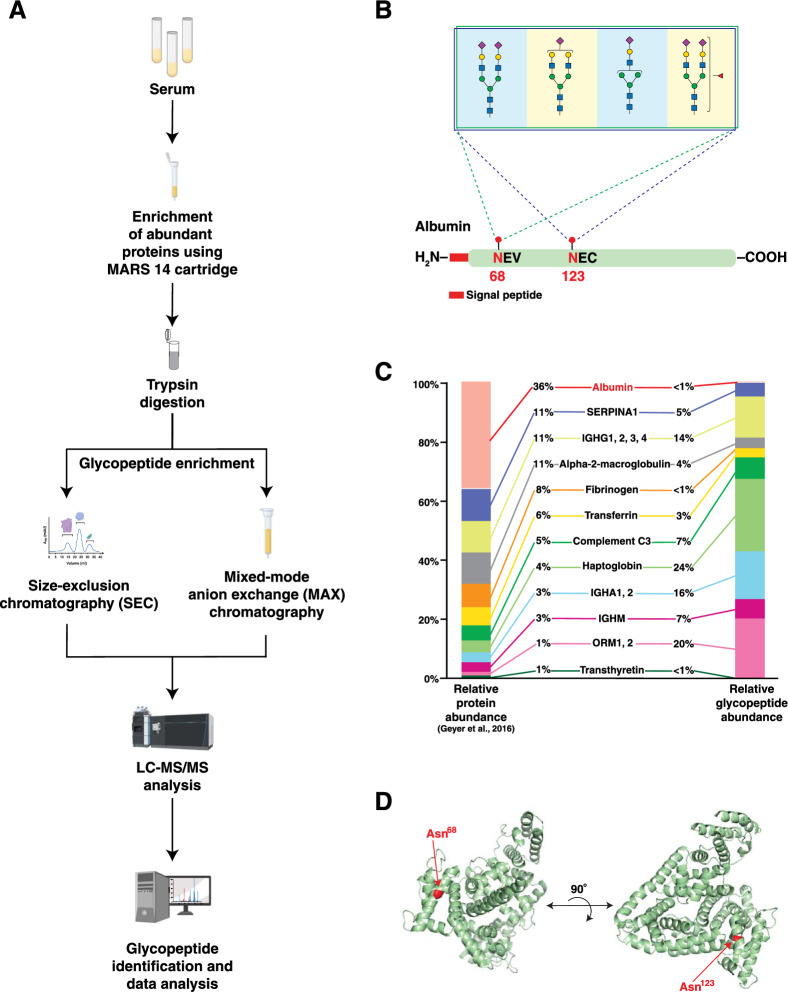
N-linked glycosylation of albumin and other abundant serum proteins. **A** Experimental strategy for discovery-based analysis of site-specific glycosylation of abundant serum proteins. **B** Representation of glycopeptides identified at Asn^68^ and Asn^123^ in human albumin with glycans identified at each site (length not drawn to scale). **C** Stacked bar charts to show relative contributions from abundant serum proteins. Relative contribution to total glycopeptide intensity from proteins enriched by MARS 14 column is plotted on the right. The relative abundance levels among the same set of proteins in plasma, i.e., at the protein level, are plotted on the left (glycopeptide data from current study; protein-level data from plasma proteomics experiments, Geyer et al., 2016) [24]. **D** Schematic representation of the crystal structure of albumin highlighting the accessible positions of the two *N*-glycosylation sites (marked in red)

For initial discovery, we analyzed serum from three volunteer donors using previously described glycoproteomic profiling methods [11]. First, we reduced the complexity of the serum glycoproteome by enriching the most abundant serum proteins using the Human 14 Multiple Affinity Removal (MARS 14) column prior to trypsin digestion. Second, we enriched glycopeptides from peptide mixtures using size-exclusion chromatography (SEC). Eight fractions from SEC were analyzed using LC–MS/MS-based discovery pipeline [11] (Fig. 1A). The resulting data were searched using pGlyco3 for glycopeptide identification [18]. The search was performed against the UniProt human proteome database and the in-built human *N*-glycan database [13]. On average, 1933 glycopeptides were detected in the three samples. The most abundant glycopeptides were from abundant serum glycoproteins including haptoglobin, alpha-1-acid glycoprotein, immunoglobulin heavy chain and complement C3. These proteins accounted for > 80% of the glycopeptide precursor peak areas. *N*-glycopeptides from albumin were detected with glycosylation at both sites Asn^68^ (LVN^68^EVTEFAK) and Asn^123^ (QEPERN^123^ECFLQHK, which contains a missed tryptic cleavage site N-terminal to the site of glycosylation). To our knowledge, this is the first report of *N*-glycosylation at Asn^123^ of albumin. At both sites, complex sialylated *N*-glycans with the following compositions were identified: Hex_5_HexNAc_4_NeuAc_2_, Hex_5_HexNAc_4_NeuAc_1_, Hex_5_HexNAc_4_NeuAc_2_Fuc_1_ and Hex_4_HexNAc_3_NeuAc_1_ (Fig. 1B). Two of these glycans, Hex_5_HexNAc_4_NeuAc_2_Fuc_1_ and Hex_4_HexNAc_3_NeuAc_1_ have not been reported previously on Asn^68^. To our surprise, albumin-derived glycopeptides accounted for < 1% of the total intensity of glycopeptides derived from abundant serum proteins even though albumin is the most abundant serum protein. The relative contribution of individual glycoproteins enriched by MARS 14 to total glycopeptide intensity from these samples is shown in Fig. 1C. We were curious to observe the relationship between the abundance of these proteins and the abundance of corresponding glycopeptides. For comparison, we used protein-level data reported by Geyer et al., 2016, to plot the relative intensities of the same proteins from plasma samples [23]. As shown in Fig. 1C, though albumin accounted for 36% total peptide share among these proteins, it only contributed 1% of the glycopeptide signal. Because *N*-glycosylation occurs more commonly on exposed regions of proteins as compared to internal, more buried regions [24], we examined the location of both glycosylation sites in the three-dimensional structure of albumin. We visualized the crystal structure of albumin from Protein Data Bank and mapped the two *N*-glycosylation sites [20]. As shown in Fig. 1D, both Asn^68^ and Asn^123^ are located on the surface of the structure of albumin.

Next, we tested an alternate strategy for glycopeptide enrichment for analysis by single MS runs. Peptides from MARS 14-enriched proteins were subjected to glycopeptide enrichment using MAX [17]. LC–MS/MS analysis of enriched samples as a single fraction led to the identification of 409 glycopeptides in each sample on average. In this method also, the most abundant serum glycoproteins described above accounted for > 80% of the glycopeptide precursor peak areas. Glycosylation at both non-canonical glycosylation sites of albumin, i.e., Asn^68^ and Asn^123^ was also detected in all three samples following MAX-enrichment. However, both sites were detected with only two glycans (Hex_5_HexNAc_4_NeuAc_2_, Hex_5_HexNAc_4_NeuAc_1_) using this method (Additional file 2: Fig. S1) Glycopeptides detected from SEC- and MAX-enriched samples are listed in Additional file 3: Tables S1 and S2, respectively.

### Relative abundance of *N*-glycans on Asn^68^ and Asn^123^

**Fig. 2:**
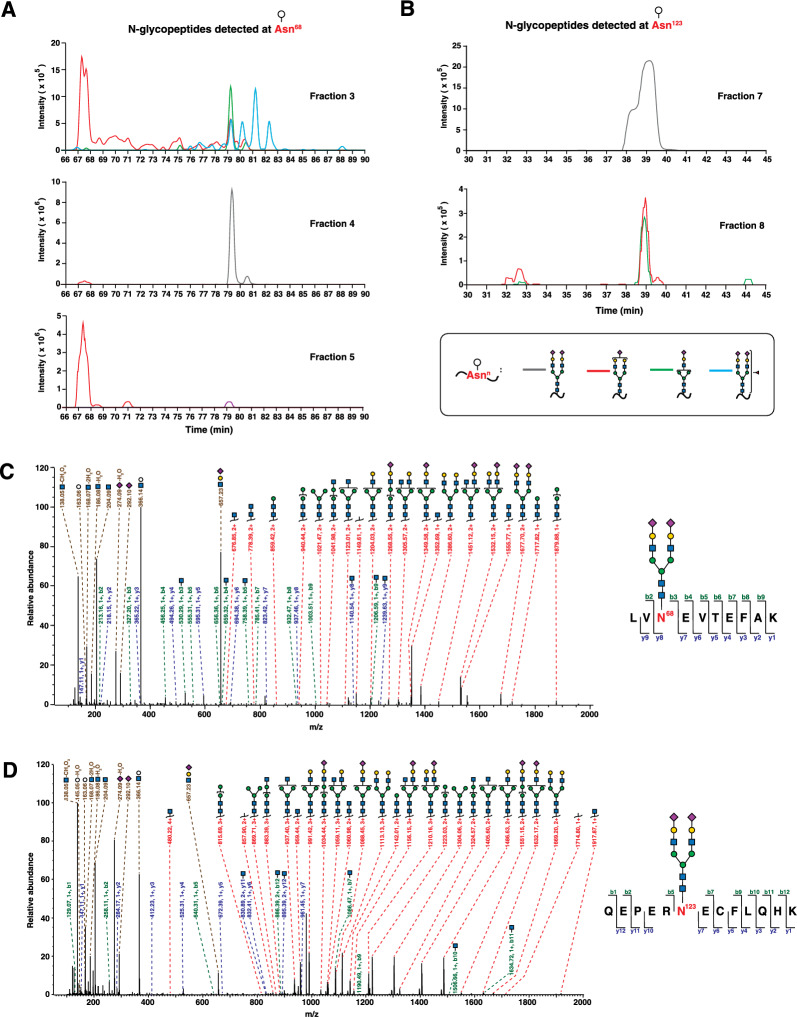
Abundance and identification of albumin-derived glycopeptides. **A** Extracted ion chromatograms (XIC) showing relative abundance of glycopeptides corresponding to Asn^68^ detected in different fractions of an individual sample from size-exclusion chromatography (SEC). In fraction 3, glycopeptides bearing the glycan Hex_5_HexNAc_4_NeuAc_2_ at this site (represented by a grey line in other fractions) were identified with peak intensity of ~ 5 × 10^7^ at 79.3 min. To clearly depict the lower-abundance glycopeptides with other compositions which would otherwise be lost to scale, we omitted the XIC of the glycopeptide bearing the glycan Hex_5_HexNAc_4_NeuAc_2_ at this site in fraction 3. **B** XICs showing the relative abundance of glycopeptides corresponding to Asn^123^ detected in different fractions of an individual sample from SEC. In fraction 8, glycopeptides bearing the glycan Hex_5_HexNAc_4_NeuAc_2_ at this site (represented by a grey line in other fractions) were identified with a peak intensity of ~ 3 × 10^7^ at 38.9 min. To clearly depict the lower-abundance glycopeptides with other compositions which would otherwise be lost to scale, we omitted the XIC of the glycopeptide bearing the glycan Hex_5_HexNAc_4_NeuAc_2_ at this site in fraction 8. **C**, **D** Annotated MS/MS fragmentation spectra of representative glycopeptides derived from albumin with the glycan Hex_5_HexNAc_4_NeuAc_2_ at sites Asn^63^ and Asn^123^, respectively

To determine the relative abundance of the glycopeptides identified from each site, we compared the peak intensity of precursor ions of the glycopeptides detected at each site in the SEC-based experiment. Glycopeptides with glycan compositions Hex_5_HexNAc_4_NeuAc_2_ and Hex_5_HexNAc_4_NeuAc_1_ were the most abundantly detected glycopeptides at both sites (Fig. 2A and B). MS/MS spectra were manually verified for evidence of oxonium ions including signature ions of sialic acid, peptide backbone ions with attached glycan fragments (Y ions) as well as fragments of the naked peptide (*b* and *y* ions) for all glycopeptides mapped to albumin. Annotated MS/MS spectra for glycopeptides from both sites are shown in Fig. 2C, D and (Additional file 2: Fig. S2A–F). These data confidently identify both Asn^68^ and Asn^123^ as *N*-glycosylation sites while also describing the microheterogeneity at each site.

### Confirmation of N-linked glycosylation sites

Next, we sought to confirm *N*-glycosylation at sites Asn^68^ and Asn^123^ of albumin by analyzing enzymatically deglycosylated peptides. Serum proteins were digested using trypsin and glycopeptides were enriched using a MAX column. Glycopeptides were treated with PNGase F using either ^16^O or ^18^O-labeled water. Deglycosylated peptides were identified considering the mass shift expected after enzymatic removal of the *N*-glycan, which is accompanied by the conversion of asparagine (Asn) to aspartic acid (Asp) [25]. Deglycosylated Asn residues were identified with conversion to Asp showing a mass difference of 0.98 Da in case of ^16^O incorporation and 2.98 Da in case of ^18^O incorporation.

**Fig. 3:**
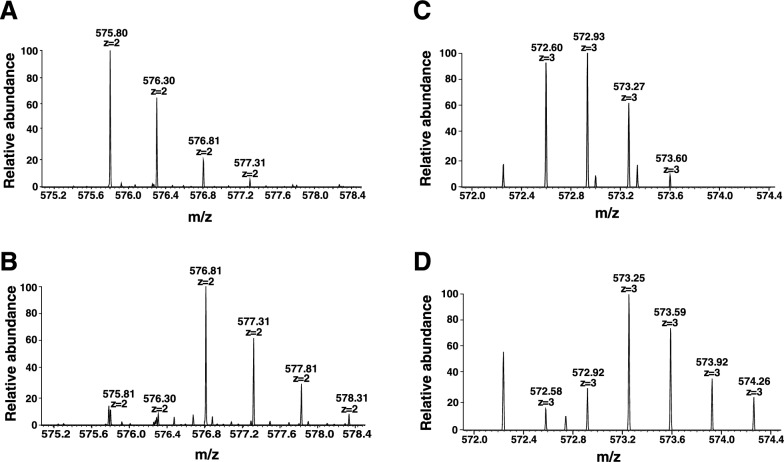
Mass spectra showing the detection of deglycosylated peptides of albumin after treatment with PNGase F in stable isotope-labeled water. **A**, **B** Precursor mass spectra of the albumin-derived glycopeptide with glycosylation at Asn^68^, detected in a charge state of +2 after deglycosylation by PNGase F treatment in the presence of H_2_^16^O with a mass shift corresponding to 0.98 Da (**A**) or in the presence of H_2_^18^O with a mass shift corresponding to 2.98 Da (**B**). **C**, **D** Precursor mass spectra of the albumin-derived glycopeptide with glycosylation at Asn^123^, detected in a charge state of +3 after deglycosylation by PNGase F treatments in H_2_^16^O with a mass shift corresponding to 0.98 Da (**C**) and in H_2_^18^O with a mass shift corresponding to 2.98 Da (**D**)

The non-glycosylated peptide with Asn^68^ (LVN^68^EVTEFAK) was identified with a charge state of + 2 with m/z of 575.31. Upon treatment with PNGase F in ^16^O water, we detected the deglycosylated form of the formerly *N*-glycosylated peptide with a mass shift of 0.98 Da or 0.5 m/z (LVD^68^EVTEFAK, m/z of 575.80, Fig. 3A). In samples treated with PNGase F in ^18^O-labeled water, we observed a mass shift of 2.98 Da or 1.5 m/z, corresponding to the deglycosylated peptide (LVD*^68^EVTEFAK, m/z of 576.81 m/z, Fig. 3B). The partial overlap of peaks from the ^16^O-labeled peptides with the ^18^O-labeled peptides is explained by the natural abundance of isotopes and purity of ^18^O-labeled water used [26] (Fig. 3B). This analysis demonstrates enzymatic deglycosylation of Asn^68^, conclusively showing albumin glycosylation at this site.

Similarly, we detected the non-glycosylated peptide containing Asn^123^ (QEPERN^123^ECFLQHK) with a charge state of +3 and m/z of 572.27. Upon treatment with PNGase F in ^16^O water, we identified the deglycosylated form of the peptide (QEPERD^123^ECFLQHK, m/z of 572.60 m/z) as depicted in Fig. 3C. With ^18^O incorporation, we observed the deamidated form QEPERD*^123^ECFLQHK at the m/z of 573.25 m/z (Fig. 3D). This confirms glycosylation at Asn^123^.

### Confirmation of albumin glycopeptides by MS3 fragmentation

**Fig. 4:**
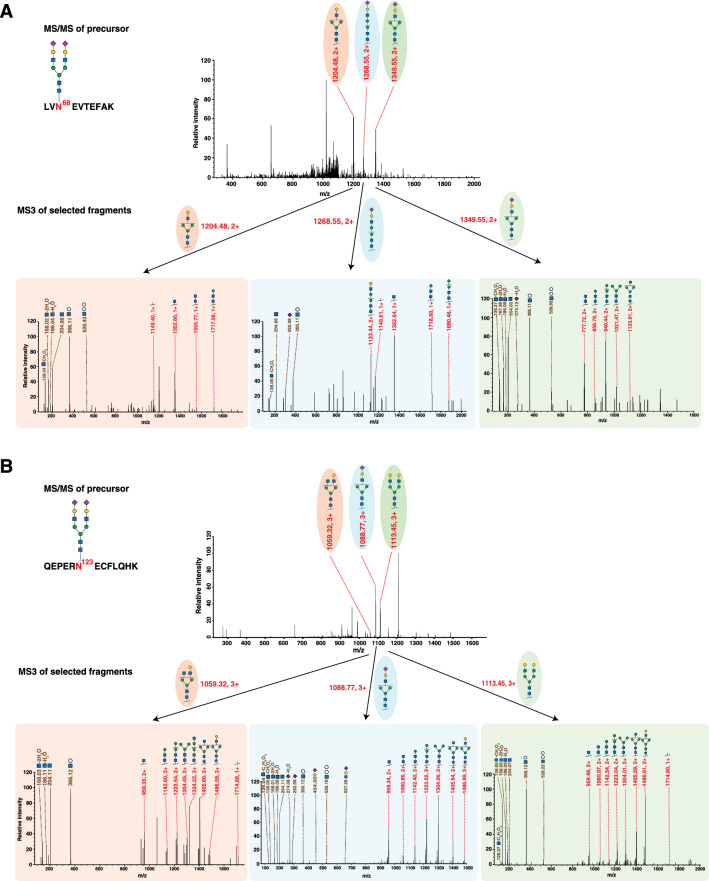
MS3 analysis of albumin-derived glycopeptides. Precursor fragmentation is shown along with fragmentation of MS/MS-derived fragments using MS3. **A** Glycopeptide LVN^68^EVTEFAK with Hex_5_HexNAc_4_NeuAc_2_ (m/z = 1118.8, charge state +3). The MS/MS fragmentation of the precursor glycopeptide is shown in the top half of the panel. The three annotated fragment Y ions (peptide backbone with glycan fragments still attached to it) from this scan were selected and further fragmented by MS3. Labeled arrows under the peak of each selected fragment ion in the MS/MS scan indicate MS3 fragmentation spectra for the corresponding Y ions. **B** Glycopeptide QEPERN^123^ECFLQHK with Hex_5_HexNAc_4_NeuAc_2_ (m/z = 980.6, charge state +4). The MS/MS fragmentation of the precursor glycopeptide is shown in the top half of the panel. The representations of the MS/MS spectrum, selected fragments and their MS3 spectra are as described in A

To further enhance the confidence in the identification of albumin-derived glycopeptides, we performed MS3 analysis using an Orbitrap Eclipse Tribrid mass spectrometer which incorporates a high-sensitivity ion-trap detector. Albumin was immunoprecipitated from pooled serum and glycopeptides were enriched by MAX. Precursor ions corresponding to four albumin-derived glycopeptides were isolated and fragmented using collision-induced dissociation (CID) followed by their detection in the ion-trap. At low collision energy, glycosidic bonds were expected to break forming ions consisting of the peptide backbone carrying glycan fragments (Y ions). Selected Y ions were fragmented at the MS3 level using higher-energy collisional dissociation (HCD) followed by detection in the ion-trap. MS3 fragmentation produced glycan oxonium ions confirming the presence of glycopeptides, as well as further fragments of the Y ions. The resulting spectra were manually inspected and annotated (Fig. 4).

The precursor ions selected included the two most abundant glycopeptides at each glycosylation site, i.e., LVN^68^EVTEFAK with Hex_5_HexNAc_4_NeuAc_1_ (m/z = 1021.7, charge state +3), LVN^68^EVTEFAK with Hex_5_HexNAc_4_NeuAc_2_ (m/z = 1118.8, charge state +3), QEPERN^123^ECFLQHK with Hex_5_HexNAc_4_NeuAc_1_ (m/z = 907.8, charge state +4), and QEPERN^123^ECFLQHK with Hex_5_HexNAc_4_NeuAc_2_ (m/z = 980.6, charge state +4). As expected, prominent product ions generated from low energy CID fragmentation at MS2 level were glycopeptide Y ions (Fig. 4). Notably, we also detected singly charged oxonium ions (albeit with lower intensity) at m/z values of 274.0 (NeuAc with water loss), 292.1 (NeuAc), 366.1 (HexNAc and Hex), and 657.2 (HexNAc, Hex, and NeuAc), further confirming the presence of glycopeptides (as depicted in Fig. 4). Subsequently, fragment Y ions for each precursor ion generated at the MS/MS level underwent further fragmentation via HCD, yielding diagnostic MS3 fragment ions. The ion series with the serial loss of single monosaccharide residues validated the glycan composition of these glycopeptides. Further, the glycan oxonium ions at the MS3 level were detected with higher intensities, confirming the presence of glycopeptides. Spectra resulting upon fragmentation of precursor ions with m/z of 1118.8 (charge state +3) and 980.6 (charge state +4) with peptide sequence and glycan composition mentioned above are shown in Fig. 4A and 4B respectively.

### Albumin glycosylation in a larger cohort of volunteer donors

To assess if glycosylation of albumin is a general phenomenon and validate our findings, we analyzed serum samples from twenty volunteer donors by targeted MS. Eight albumin-derived glycopeptides identified in the discovery experiment were targeted, i.e., glycopeptides with sequences LVN^68^EVTEFAK and QEPERN^123^ECFLQHK, each bearing one of four glycans, Hex_5_HexNAc_4_NeuAc_2_, Hex_5_HexNAc_4_NeuAc_1_, Hex_5_HexNAc_4_NeuAc_2_Fuc_1_ and Hex_4_HexNAc_3_NeuAc_1_. MAX-enriched *N*-glycopeptides from serum proteins were analyzed by parallel reaction monitoring-mass spectrometry (PRM-MS). In all the twenty individuals that were tested, we detected glycosylation at both Asn^68^ and Asn^123^ of albumin. The heterogeneity in the overall glycopeptide complement detected among the individuals is shown in Table S3 (Additional file 3).

### Albumin glycosylation in other species

**Fig. 5:**
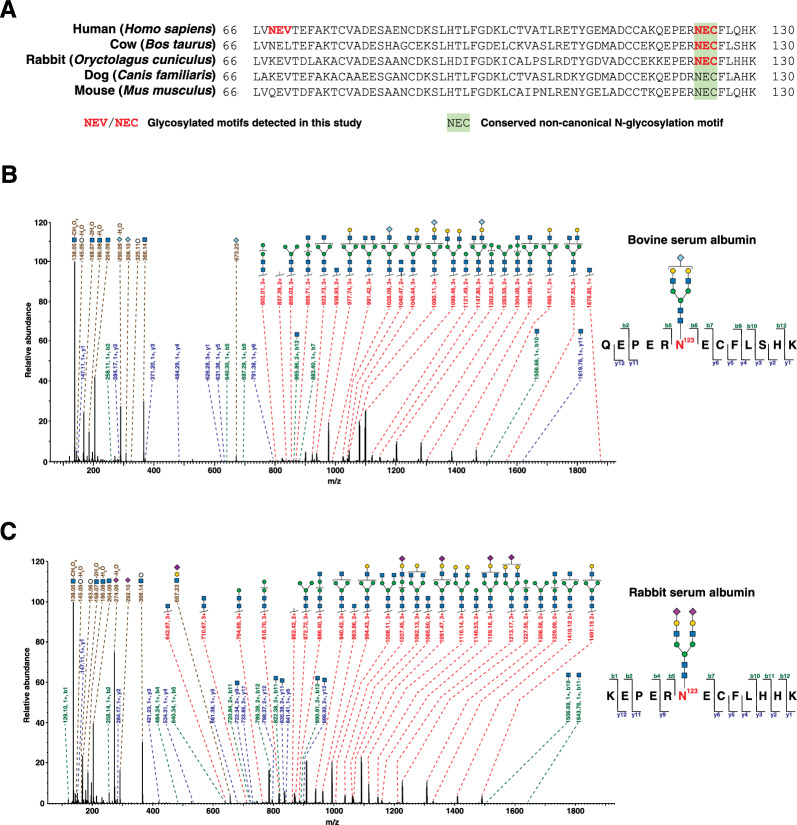
Glycosylation of albumin orthologs in other mammalian species.** A** Multiple sequence alignment of the region of human albumin containing the two *N*-glycosylation sites with orthologs from selected mammalian species. The conserved non-canonical *N*-glycosylation motif with Asn^123^ is shown highlighted in green. Non-canonical *N*-glycosylation motifs that were detected with glycosylation in this study are shown in red font. **B** Annotated MS/MS fragmentation spectrum of glycopeptide derived from bovine serum albumin (BSA) at site Asn^123^ with the glycan Hex_5_HexNAc_4_NeuGc_1_. **C** Annotated MS/MS fragmentation spectrum of glycopeptide derived from rabbit serum albumin at site Asn^123^ with the glycan Hex_5_HexNAc_4_NeuAc_2_, with annotations as described in **B**

Because albumin is a highly conserved protein, we were curious if its orthologs in other mammalian species are also glycosylated. Examining the amino acid sequences of albumin orthologs from cow, rabbit, dog and mouse revealed that only albumin from mouse has canonical Asn-X-Ser/Thr motifs, but without annotation for N-linked glycosylation on UniProt [13]. However, these orthologs have multiple non-canonical *N*-glycosylation motifs. Multiple sequence alignment showed that the non-canonical motif Asn^123^-Glu-Cys, is highly conserved, whereas site Asn^68^ is not an evolutionarily conserved glycosylation site or amino acid (Fig. 5A). Therefore, to test if this site is glycosylated in other species, we analyzed bovine serum albumin (BSA) and rabbit serum albumin, which are commonly used in molecular biology and MS applications. Commercially available BSA was digested using trypsin followed by MAX-based enrichment of glycopeptides followed by LC–MS/MS analysis for glycopeptide discovery. Database searching for glycopeptides was done using pGlyco3 with the UniProt bovine proteome database for peptide sequences. As bovine *N*-glycans are similar in composition to human *N*-glycans except for the presence of an additional sialic acid (*N*-glycolylneuraminic acid or NeuGc) which is also present in mouse, we used the in-built mouse *N*-glycan database for this search [27]. We detected BSA-derived glycopeptides with Asn^123^ glycosylated by three complex sialylated glycans, i.e., Hex_5_HexNAc_4_NeuGc_1_, Hex_5_HexNAc_4_NeuAc_1_ and Hex_5_HexNAc_4_NeuAc_1_NeuGc_1_ (Fig. 5, Additional file 2: Fig. S3A and S3B respectively). Interestingly, besides glycosylation at the conserved site Asn^123^, we also detected glycopeptides from BSA with glycosylation at Asn^185^ with two glycans, Hex_5_HexNAc_4_NeuAc_2_ and Hex_5_HexNAc_4_NeuAc_1_NeuGc_1_ (Additional file 2: Fig. S3C and S3D respectively). However, this non-canonical glycosylation site, which is in the motif Asn^185^-Gly-Val, is not conserved across the species listed above. Additionally, in a separate experiment performed identically but with commercially available rabbit serum albumin and searched against the rabbit proteome and mouse *N*-glycan database, the conserved non-canonical *N*-glycosylation site Asn^123^ was also detected with two complex sialylated *N*-glycans, i.e., Hex_5_HexNAc_4_NeuAc_2_ and Hex_5_HexNAc_4_NeuAc_1_ (Fig. 5C; Additional file 2: Fig. S3E respectively). Overall, these data provide evidence for the glycosylation of albumin at the conserved non-canonical *N*-glycosylation site orthologous to Asn^123^ of human albumin in two additional mammalian species. Glycopeptides detected in bovine and rabbit serum albumin are listed in Additional file 3: Tables S4 and S5 respectively.

## Discussion

Although most abundant serum proteins are glycoproteins, albumin itself has been considered a notable exception until recently [10]. Through discovery analysis and rigorous testing using different enrichment strategies [11, 15] and high-resolution LC–MS/MS methods, we report a novel *N*-glycosylation site on albumin (Asn^123^) and expand the glycan heterogeneity on another site (Asn^68^). Effective enrichment strategies are key to MS-based identification of glycopeptides owing to glycan heterogeneity [15, 28]. In the discovery experiments, SEC, which is based on physical properties and used here as a method for simultaneous enrichment and fractionation, resulted in identification of three times more glycopeptides in comparison to the single MS runs after MAX-based enrichment. Albumin glycopeptides at sites Asn^68^ and Asn^123^ were identified by both methods. Interestingly, though albumin is the most abundant plasma protein, glycopeptides from albumin accounted for < 1% of identified glycopeptide precursor peak areas, indicating low site occupancy (Fig. 1C). This follows our expectation based on previous reports on other proteins that non-canonical *N*-glycosylation motifs have lower stoichiometry of glycosylation [8, 9]. We also show that Asn^123^, which occurs within a highly conserved Asn-Glu-Cys motif is also glycosylated in bovine and rabbit serum albumin. In the case of BSA, we detected two glycopeptides containing NeuGc, a sialic acid that is not present in humans because the gene encoding an essential synthetic enzyme, cytidine monophosphate-*N*-acetylneuraminic acid hydroxylase (*CMAH*), is inactive in humans [29]. Though BSA is routinely used as a tool for quality control for MS, we believe that its glycosylation has generally been missed previously because of the absence of suspicion owing to lack of a consensus *N*-glycosylation motif.

Physiologically, albumin is involved in several functions including binding and transportation of molecules such as fatty acids, hormones, drugs, vitamins and metal ions [30, 31]. These ligand-binding and antioxidant functions of albumin are influenced by its various post-translational modifications (PTMs) [30] including cysteinylation, oxidation and nitrosylation [31]. Additionally, glycation is present at 20–30% in circulating albumin in hyperglycemic individuals, and this modification alters its binding properties [2, 32]. Traditional methods of protein analysis, e.g., isoelectric focusing (IEF) and two-dimensional gel electrophoreses (2DE) did not raise any suspicions of glycosylation of albumin on record, even though some such studies report separation of albumin into fractions based on isoelectric point [33]. In light of the current report, we wonder if the smears and unexplained spots annotated for albumin on IEF and 2DE experiments may be explained, at least in part, by albumin *N*-glycoforms [33, 34]. Additional studies may determine functional effects of glycosylation on the ligand-binding and antioxidant properties of albumin, along with its susceptibility to undergo other PTMs [35]. For example, Cys^125^, which is the C-terminal amino acid in the motif that Asn^123^ is part of (Asn^123^-Glu^124^-Cys^125^), participates in the formation of a disulfide bridge in the secondary structure of albumin [36]. It has been previously shown that degree of glycosylation at sites in Asn-X-Cys motifs is likely related to the rate of translation as well as the rate of disulfide bond formation [7]. Hence, the rate of glycosylation at Asn^123^ may be altered in states such as liver disease and metabolic syndrome where liver function is affected [37].

## Conclusions

To conclude, we report that albumin is a glycoprotein with multiple N-linked glycoforms at two non-canonical sites. As these findings are discordant with the long-held notion that albumin is a non-glycosylated protein, we confirmed them by multiple additional lines of investigation. Serum albumin level is used as a marker for several diseases including renal, hepatic and cardiovascular disorders [38]. Pathological modifications of albumin including glycation and cysteinylation are also associated with diabetes and liver disease [39]. In fact, glycated albumin has been shown to complement glycated hemoglobin as a marker of prediabetes [40]. Given this importance of albumin in clinical practice, glycosylated albumin could also have clinical significance. Indeed, we have recently found reduced levels of the glycopeptide bearing Hex_5_HexNAc_4_NeuAc_1_ at Asn^123^ in patients with a congenital disorder of glycosylation (CDG) [41]. This indicates that glycosylation events on albumin could potentially be of diagnostic or other clinical uses. Future studies may determine the exact role of glycosylation of albumin and how it is altered in other diseases associated with altered protein glycosylation. Our findings alter the prevailing paradigm by showing that albumin is not a non-glycosylated protein and may expand our understanding of its structure and function, and its clinical and biochemical applications.

### Supplementary Information

**Additional file 1: Supplemental Methods.** Additional experimental details, materials and methods.

**Additional file 2: Additional figures.** Additional supporting figures providing additional information on the glycopeptides identified by discovery analysis of human serum, bovine serum albumin and rabbit serum albumin samples.

**Additional file 3: Additional Tables.** Additional information on glycopeptides identified by SEC- and MAX-based enrichment; albumin-derived glycopeptides identified in additional volunteer donor samples; glycopeptides identified from bovine and rabbit serum albumin samples.

## Data Availability

The mass spectrometry proteomics data have been deposited to the ProteomeXchange Consortium via the PRIDE [43] partner repository with the dataset identifier PXD047863.

## Abbreviations

MARS 14: Multiple affinity removal spin cartridge human-14
SEC: Size-exclusion chromatography
MAX: Mixed-mode anion exchange
LC–MS/MS: Liquid chromatography–tandem mass spectrometry
PRM: Parallel reaction mode
Asn^x^: Asparagine at amino acid site x in a polypeptide sequence
PTM: Post-translational modification
MS: Mass spectrometry
TEABC: Triethylammonium bicarbonate
DTT: Dithiothreitol
IAA: Iodoacetamide
TFA: Trifluoroacetic acid
FA: Formic acid
ACN: Acetonitrile
DDA: Data-dependent analysis
AGC: Automatic gain control
HCD: Higher-energy collisional dissociation
FDR: False-discovery rate
MS/MS: Tandem mass spectrometry
BSA: Bovine serum albumin
PDB: Protein Data Bank
PBS: Phosphate-buffered saline
CID: Collision-induced dissociation
Hex: Hexose
HexNAc: *N*-Acetylhexosamine
NeuAc: *N*-Acetylneuraminic acid
Fuc: Fucose
NeuGc: *N*-Glycolylneuraminic acid
SNFG: Graphical representations of glycans are made using Symbol Nomenclature for Glycans [42]

## References

[1] Spiro RG (2002). Protein glycosylation: nature, distribution, enzymatic formation, and disease implications of glycopeptide bonds. Glycobiology.

[2] Rondeau P, Bourdon E (2011). The glycation of albumin: structural and functional impacts. Biochimie.

[3] Schjoldager KT, Narimatsu Y, Joshi HJ, Clausen H (2020). Global view of human protein glycosylation pathways and functions. Nat Rev Mol Cell Biol.

[4] Stanley P, Moremen KW, Lewis NE, Taniguchi N, Aebi M. N-Glycans. In: Varki A, Cummings RD, Esko JD, Stanley P, Hart GW, Aebi M, et al., editors. Essentials of Glycobiology. 4th ed. Cold Spring Harbor (NY) 2022, 103–16.

[5] Bause E, Legler G (1981). The role of the hydroxy amino acid in the triplet sequence Asn-Xaa-Thr(Ser) for the N-glycosylation step during glycoprotein biosynthesis. Biochem J.

[6] Hulsmeier AJ, Tobler M, Burda P, Hennet T (2016). Glycosylation site occupancy in health, congenital disorder of glycosylation and fatty liver disease. Sci Rep.

[7] Lowenthal MS, Davis KS, Formolo T, Kilpatrick LE, Phinney KW (2016). Identification of novel N-glycosylation sites at noncanonical protein consensus motifs. J Proteome Res.

[8] Canis K, McKinnon TA, Nowak A, Haslam SM, Panico M, Morris HR (2012). Mapping the N-glycome of human von Willebrand factor. Biochem J.

[9] Satomi Y, Shimonishi Y, Takao T (2004). N-glycosylation at Asn(491) in the Asn-Xaa-Cys motif of human transferrin. FEBS Lett.

[10] Sun S, Hu Y, Jia L, Eshghi ST, Liu Y, Shah P (2018). Site-specific profiling of serum glycoproteins using N-linked glycan and glycosite analysis revealing atypical N-glycosylation sites on albumin and alpha-1B-glycoprotein. Anal Chem.

[11] Saraswat M, Garapati K, Mun DG, Pandey A (2021). Extensive heterogeneity of glycopeptides in plasma revealed by deep glycoproteomic analysis using size-exclusion chromatography. Mol Omics.

[12] Zielinska DF, Gnad F, Wisniewski JR, Mann M (2010). Precision mapping of an in vivo N-glycoproteome reveals rigid topological and sequence constraints. Cell.

[13] UniProt C (2021). UniProt: the universal protein knowledgebase in 2021. Nucleic Acids Res.

[14] Quinlan GJ, Martin GS, Evans TW (2005). Albumin: biochemical properties and therapeutic potential. Hepatology.

[15] Riley NM, Bertozzi CR, Pitteri SJ (2021). A pragmatic guide to enrichment strategies for mass spectrometry-based glycoproteomics. Mol Cell Proteomics.

[16] Budhraja R, Saraswat M, De Graef D, Ranatunga W, Ramarajan MG, Mousa J (2023). N-glycoproteomics reveals distinct glycosylation alterations in NGLY1-deficient patient-derived dermal fibroblasts. J Inherit Metab Dis.

[17] Yang W, Shah P, Hu Y, Toghi Eshghi S, Sun S, Liu Y (2017). Comparison of enrichment methods for intact N- and O-linked glycopeptides using strong anion exchange and hydrophilic interaction liquid chromatography. Anal Chem.

[18] Zeng WF, Cao WQ, Liu MQ, He SM, Yang PY (2021). Precise, fast and comprehensive analysis of intact glycopeptides and modified glycans with pGlyco3. Nat Methods.

[19] Sugio S, Kashima A, Mochizuki S, Noda M, Kobayashi K (1999). Crystal structure of human serum albumin at 2.5 A resolution. Protein Eng.

[20] Berman HM, Battistuz T, Bhat TN, Bluhm WF, Bourne PE, Burkhardt K (2002). The protein data bank. Acta Crystallogr D Biol Crystallogr.

[21] Schrodinger, LLC. The PyMOL molecular graphics system, Version 1.8. 2015.

[22] Pino LK, Searle BC, Bollinger JG, Nunn B, MacLean B, MacCoss MJ (2020). The Skyline ecosystem: informatics for quantitative mass spectrometry proteomics. Mass Spectrom Rev.

[23] Geyer PE, Kulak NA, Pichler G, Holdt LM, Teupser D, Mann M (2016). Plasma proteome profiling to assess human health and disease. Cell Syst.

[24] Petrescu AJ, Milac AL, Petrescu SM, Dwek RA, Wormald MR (2004). Statistical analysis of the protein environment of N-glycosylation sites: implications for occupancy, structure, and folding. Glycobiology.

[25] Kuster B, Mann M (1999). 18O-labeling of N-glycosylation sites to improve the identification of gel-separated glycoproteins using peptide mass mapping and database searching. Anal Chem.

[26] Kaji H, Saito H, Yamauchi Y, Shinkawa T, Taoka M, Hirabayashi J (2003). Lectin affinity capture, isotope-coded tagging and mass spectrometry to identify N-linked glycoproteins. Nat Biotechnol.

[27] Nwosu CC, Aldredge DL, Lee H, Lerno LA, Zivkovic AM, German JB (2012). Comparison of the human and bovine milk N-glycome via high-performance microfluidic chip liquid chromatography and tandem mass spectrometry. J Proteome Res.

[28] Bagdonaite I, Malaker SA, Polasky DA, Riley NM, Schjoldager K, Vakhrushev SY (2022). Glycoproteomics. Nat Rev Methods Primers.

[29] Chou HH, Takematsu H, Diaz S, Iber J, Nickerson E, Wright KL (1998). A mutation in human CMP-sialic acid hydroxylase occurred after the Homo-Pan divergence. Proc Natl Acad Sci USA.

[30] Fasano M, Curry S, Terreno E, Galliano M, Fanali G, Narciso P (2005). The extraordinary ligand binding properties of human serum albumin. IUBMB Life.

[31] Rahali MA, Lakis R, Sauvage FL, Pinault E, Marquet P, Saint-Marcoux F (2023). Posttranslational-modifications of human-serum-albumin analysis by a top-down approach validated by a comprehensive bottom-up analysis. J Chromatogr B Analyt Technol Biomed Life Sci.

[32] Fanali G, di Masi A, Trezza V, Marino M, Fasano M, Ascenzi P (2012). Human serum albumin: from bench to bedside. Mol Aspects Med.

[33] Chromy BA, Gonzales AD, Perkins J, Choi MW, Corzett MH, Chang BC (2004). Proteomic analysis of human serum by two-dimensional differential gel electrophoresis after depletion of high-abundant proteins. J Proteome Res.

[34] Ong SE, Pandey A (2001). An evaluation of the use of two-dimensional gel electrophoresis in proteomics. Biomol Eng.

[35] Zacchi LF, Schulz BL (2016). N-glycoprotein macroheterogeneity: biological implications and proteomic characterization. Glycoconj J.

[36] Bocedi A, Cattani G, Stella L, Massoud R, Ricci G (2018). Thiol disulfide exchange reactions in human serum albumin: the apparent paradox of the redox transitions of Cys(34). FEBS J.

[37] Levitt DG, Levitt MD (2016). Human serum albumin homeostasis: a new look at the roles of synthesis, catabolism, renal and gastrointestinal excretion, and the clinical value of serum albumin measurements. Int J Gen Med.

[38] Ballmer PE (2001). Causes and mechanisms of hypoalbuminaemia. Clin Nutr.

[39] Domenicali M, Baldassarre M, Giannone FA, Naldi M, Mastroroberto M, Biselli M (2014). Posttranscriptional changes of serum albumin: clinical and prognostic significance in hospitalized patients with cirrhosis. Hepatology.

[40] Sumner AE, Duong MT, Bingham BA, Aldana PC, Ricks M, Mabundo LS (2016). Glycated albumin identifies prediabetes not detected by hemoglobin A1c: the Africans in America Study. Clin Chem.

[41] Garapati K, Budhraja R, Saraswat M, Kim J, Joshi N, Sachdeva GS, et al. A complement C4-derived glycopeptide as a biomarker for PMM2-CDG. JCI Insight. 2024;In Press. 10.1172/jci.insight.172509 PMC7615924 38587076

[42] Neelamegham S, Aoki-Kinoshita K, Bolton E, Frank M, Lisacek F, Lutteke T (2019). Updates to the symbol nomenclature for Glycans guidelines. Glycobiology.

[43] Perez-Riverol Y, Bai J, Bandla C, Garcia-Seisdedos D, Hewapathirana S, Kamatchinathan S (2022). The PRIDE database resources in 2022: a hub for mass spectrometry-based proteomics evidences. Nucleic Acids Res.

