# Supplementary material for: Defining albumin as a glycoprotein with multiple N-linked glycosylation sites
Source: J Transl Med. 2024 May 13;22:454. doi: 10.1186/s12967-024-05000-5 (PMC11090807; doi:10.1186/s12967-024-05000-5)
Supplement: Supplementary file 1 — Additional file 1: Supplemental Methods. Additional experimental details, materials and methods. [file 12967_2024_5000_MOESM1_ESM.pdf]

Additional File 1

Defining albumin as a glycoprotein with multiple N-linked glycosylation sites

Kishore Garapati[1,2,3,*], Anu Jain[3,*], Benjamin J. Madden[4], Dong-Gi Mun[3], Jyoti Sharma[1,2], Rohit Budhraja[3], Akhilesh Pandey[3,5,§]

[1]Manipal Academy of Higher Education (MAHE), Manipal, Karnataka, India

[2]Institute of Bioinformatics, International Technology Park, Bangalore, Karnataka, India

[3]Department of Laboratory Medicine and Pathology, Mayo Clinic, Rochester, Minnesota, USA

[4]Proteomics Core, Mayo Clinic, Rochester, Minnesota, USA

[5]Center for Individualized Medicine, Mayo Clinic, Rochester, Minnesota, United States

*These authors contributed equally

§Corresponding Author

Akhilesh Pandey, M.D., Ph.D.

Department of Laboratory Medicine and Pathology

Mayo Clinic

200 First Street SW

Rochester, MN 55905, USA

Tel: +1-507-293-9564

Email: pandey.akhilesh@mayo.edu

Supplemental Methods

Samples

Twenty-three serum samples used in this study were deidentified residual samples from volunteer donors (approved by Mayo Clinic IRB: 21-012890).

Enrichment of abundant serum proteins

Serum samples from volunteer donors were first enriched for 14 most-abundant serum proteins using multiple affinity removal spin cartridge human 14 (MARS 14; Agilent, catalog number 5188-6560) following manufacturer's instructions. Briefly, MARS 14 cartridges were first equilibrated with manufacturer-supplied buffer A (Agilent, catalog number 5185-5987) followed by loading of serum samples on the cartridges. The cartridges were spun at low speed for a minute. The cartridges were washed twice with buffer A and cartridge-bound fraction was eluted by passing manufacturer supplied buffer B (Agilent, catalog number 5185-5988) through the cartridges. Buffer B was removed from the bound fraction by buffer exchange with 100 mM triethyl ammonium bicarbonate (prepared from Sigma TEABC, catalog number T7408). For this, the bound fraction was passed through a 5 kDa molecular weight cut-off spin filter at a speed of 5000 x g to concentrate the proteins followed by washing with 100 mM TEABC. Total protein amount was estimated using bicinchoninic acid assay and equal amounts of protein from each sample were subjected to proteolytic digestion and glycopeptide enrichment.

Protein digestion

Proteins enriched using MARS 14 cartridges were reduced and alkylated with dithiothreitol (DTT, ≥98%, Sigma, catalog number 0632) and iodoacetamide (IAA, >99%, Sigma, catalog number I1149), respectively. Trypsin (93% protein, Worthington, catalog number LS003744) was added to the proteins (1:20) and incubated overnight at 37 ºC. After digestion, the samples were acidified to a final concentration of 1% trifluoroacetic acid (prepared from Pierce TFA, 99.5%, catalog number 28904) followed by desalting using C_{18} cartridges (SepPak, Waters, catalog number WAT023501). The eluate was dried down in a speed vacuum system and peptide quantitation was performed by a colorimetric peptide assay (Thermo Scientific, catalog number 23275) prior to glycopeptide enrichment. Commercially available bovine (Thermo Scientific, catalog number 23208) and rabbit (~96%, Sigma, catalog number A9438) serum albumin were processed in a similar fashion to obtain peptides prior to glycopeptide enrichment using mixed-mode anion exchange chromatography (MAX) as described below.

Glycopeptide enrichment using size exclusion chromatography

Glycopeptides were enriched from the peptide mixture using size exclusion chromatography (SEC) as described earlier (1). Four mg of dried peptides from each sample were reconstituted in 100 µl of 0.1% formic acid (prepared from Thermo Scientific FA, >99%, catalog number 28905). Peptides were loaded and separated onto Superdex peptide 10/300 column (Cytiva, catalog number 29219757). Isocratic flow with 0.1% formic acid (FA) at a rate of 200 µl per min was used for a run time of 130 min and 48 fractions were collected. Based on the UV profile, early 16 fractions

starting from 45 min to 85 min were collected and concatenated to make 8 fractions which were dried down and reconstituted in 0.1% FA for LC-MS/MS analysis.

Glycopeptide enrichment using mixed mode anion exchange cartridges

OASIS mixed mode anion exchange cartridges (MAX, Waters, catalog number 186000366) were used in parallel with SEC for glycopeptide enrichment using previously described protocols (2). Briefly, 600 μg of dried peptides were resuspended in 95% ACN/1% TFA (prepared from Thermo Fisher ACN, ≥99.5%, catalog number A996 and Pierce TFA, 99.5%, catalog number 28904) and cartridges were conditioned three times each with acetonitrile (ACN), 100 mM triethylammonium acetate buffer, water and then 95% ACN/1% TFA. Peptides were loaded onto the cartridges followed by three washes with 95% ACN/1% TFA. Bound glycopeptides were eluted with 50% ACN/0.1% TFA and dried down in a speed vacuum system. Dried glycopeptides were reconstituted in 0.1% formic acid (FA) for LC-MS/MS analysis.

LC-MS/MS analysis in data dependent acquisition mode

Glycopeptides were analyzed by LC-MS/MS using previously described methods with modifications (1). Briefly, glycopeptides were separated using an Ultimate 3000 (Thermo Fisher Scientific) liquid chromatography system and analyzed on an Orbitrap Eclipse mass spectrometer (Thermo Fisher Scientific Inc.). Glycopeptides were first trapped on a trap column (100 mm × 2 cm, Acclaim PepMap100 Nano-Trap, Thermo Fisher Scientific) at a flow rate of 5 µl/min and separated on an EASY-Spray analytical column (75 μm x 50 cm, PepMap RSLC C_{18}, Thermo

Fisher Scientific) packed with 2 μm C_{18} particles with temperature maintained at 50 ºC. Separation was performed using 0.1% formic acid in water as solvent A and 0.1% formic acid in can as solvent B. Flow rate was maintained at 300 nl/min for a gradient time of 150 min as follows: 3% to 35% of solvent B from 4 to 130 min, then 35% to 80% of solvent B from 130 to 135 min followed by 80% of solvent B for 10 min and finally equilibration for 5 min at 3% of solvent B.

Data acquisition was done in a data-dependent acquisition (DDA) mode with the following parameters. Precursor ions were detected in the Orbitrap at a resolution of 120,000 with a scan range of 375 to 2,000 m/z with automatic gain control (AGC) target of 100% and maximum ion injection time of 50 ms. Precursor ions were isolated at a window of 1.2 m/z with charge states ranging from +2 to +7 for MS/MS events. Fragmentation was done using normalized stepped higher-energy collisional dissociation (HCD) method using normalized energies of 15, 25 and 40%. AGC was set to 400% with a maximum ion injection time of 200 ms for MS/MS. Data acquisition was performed in centroid mode with option of lock mass (441.1200025 m/z).

Database searching and analysis

Data from experiments with human samples were searched against the UniProt human reviewed protein sequences (20,432 entries, downloaded February 1, 2021) in pGlyco3 (publicly available). Default human N-glycan database (available with the software) was used. Enzyme specificity was set to trypsin with up to 2 missed cleavages and precursor and fragment tolerance was set to 10 and 20 ppm respectively. Carbamidomethylation of cysteine and oxidation of methionine were set as static and variable modifications respectively and the results were filtered for 1% false discovery

rate (FDR) at the glycopeptide level. Glycopeptide identities for albumin-derived glycopeptides were also verified by manual inspection and annotation of MS/MS spectra. Glycopeptide spectral matches were consolidated to reflect only unique glycopeptides by peptide sequence and glycan composition and monoisotopic areas were summed up to calculate the abundance of each glycopeptide. Data from the discovery analysis of bovine and rabbit serum albumin-derived glycopeptides were searched in a similar fashion in pGlyco3. For protein sequences, the BSA sample was searched against the UniProt bovine proteome and rabbit serum albumin sample was searched against the UniProt rabbit proteome. For glycans, both bovine and rabbit serum albumin samples were searched against an in-built mouse glycan database which includes glycans that are known to be present in bovine and rabbit glycoproteins but are absent in human glycoproteins. Data analysis and interpretation was done as described above.

Mapping N-glycosylation sites onto structure of albumin

The protein sequence of mature human albumin was downloaded from the UniProt database (identifier P02768) (3) and a BLASTP alignment of human albumin protein sequence was performed against Protein Data Bank (PDB). The sequence corresponding to the crystal structure of human albumin derived from pooled human plasma with the identifier 1AO6 (4) was found to be identical. The structure coordinates of 1AO6 were obtained from the PDB (5) and the structure of albumin was visualized using PyMOL (v2.5.7) (6). N-linked glycosylation site Asn^{68} was highlighted in red color. The structure was rotated by 90° to visualize the other glycosylation site, Asn^{123}, which was also highlighted in red.

Deglycosylation analysis of serum glycoproteins

Serum proteins were digested with trypsin and desalted using C_{18} cartridges (SepPak, Waters, catalog number WTWAT036820) as described above. Glycopeptides obtained after MAX enrichment were treated overnight with PNGase F (>95%, N-Zyme Scientifics, PRIME-LY™) in the presence of either ^{16}O or ^{18}O water (97% ^{18}O enriched, Sigma, catalog number 329878-1G) at 37ºC. Deglycosylated peptides were desalted using C_{18} cartridges (SepPak, Waters, catalog number WTWAT036820) and quantified by a colorimetric peptide assay (Thermo Scientific, catalog number 23275). These deglycosylated peptides were analyzed by mass spectrometry on an Orbitrap Exploris 480 (Thermo Fisher Scientific Inc.) coupled with Ultimate 3000 (Thermo Fisher Scientific Inc.) liquid chromatography system over a 75 min run time in parallel reaction monitoring mode. The gradient used was as follows: 5% of solvent B for first 4 min, 5 to 40% from 4 min to 45 min, 40 to 90% from 45 to 60 min followed by washing at 90% solvent B for 10 min and equilibration with 3% of solvent B for 5 min. The peptides were first trapped on a trap column (13.5 mm × 180µm, 2.7µm, $C_{18,}$ Optimize Technologies) at a flow rate of 5 µl/min and then separated on a PepSep analytical column (75 µm x 40 cm, C_{18}). Precursor ions were detected in the Orbitrap at a resolution of 120,000 with a scan range of 350 m/z to 1,500 m/z with AGC target of 100% and maximum ion injection time of 50 ms. MS/MS spectra were obtained at a resolution of 15000 with normalized HCD at 28%. Spectral inspection and peak identification were done manually.

Immunoprecipitation of albumin

Serum samples were incubated with anti-albumin antibody (Invitrogen, catalog number MA5-29022) in a microcentrifuge tube overnight at 4ºC with rotation. Next day, protein A-Sepharose beads (Invitrogen, catalog number 101042) were washed three times by centrifugation at 700 x g for 1 min in a spin column with ice-cold phosphate buffered saline (PBS). Protein and antibody complex was added to the spin column containing the protein A agarose beads and incubated at 4ºC with rotation for 2 hours. After incubation, the unbound fraction was collected by centrifugation at 700 x g for 1 min and the beads were washed three times with ice-cold PBS. Bound proteins were reduced with DTT and IAA followed by on-bead digestion with trypsin overnight at 37 ºC. Peptides were collected by centrifugation and desalted using C_{18} cartridges followed by MAX-enrichment of glycopeptides. Glycopeptides were analyzed by mass spectrometry using MS3 methods as described below.

MS3 analysis

Selected glycopeptides were analyzed in the MS3 mode on an Orbitrap Eclipse mass spectrometer. Glycopeptides were separated by liquid chromatography system using the same parameters and gradient time as described for DDA analysis. Precursor ions were detected in the Orbitrap at a resolution of 120,000 with a scan range of 800 to 1500 m/z. Precursor ions 1021.7 (charge state +3), 1118.8 (charge state +3), 907.8 (charge state +4) and 980.6 (charge state +4) were selected and fragmented in the ion-trap using collision induced dissociation (CID) at 40% with an activation time of 10 ms and q value of 0.25. AGC target was set to 200% and maximum ion injection time was 100 ms. Fragment ions were detected in the ion-trap and selected fragment ions for each

precursor were further fragmented using HCD at 27%. Data was analyzed and fragment ions were annotated in the MS2 and MS3 spectra manually.

Targeted LC-MS/MS analysis

Glycopeptides were analyzed in targeted mode on an Orbitrap Exploris 480 (Thermo Fisher Scientific Inc.) coupled with Ultimate 3000 liquid chromatography system. Glycopeptides were first trapped on a trap column (13.5 mm × 180μm, 2.7μm, C_{18}) at a flow rate of 5 µl/min and separated on a PepSep analytical column (75 μm x 40 cm, C_{18}). Gradient time was 85 min, and the gradient was as follows: 5% for first 4 min and 5 to 40% solvent B from 4 min to 60 min followed by washing at 90% solvent B from 70 min to 80 min and equilibration at 3% solvent B for 5 min. Precursor ions were detected in the Orbitrap at a resolution of 120,000 with a scan range of 800 m/z to 1500 m/z with AGC target of 100% and maximum ion injection time of 50 ms. MS/MS spectra were obtained at a resolution of 15000 with normalized stepped HCD at 15%, 25% and 40%. Inclusion list consisted of precursor ions for all the detected albumin glycopeptides. Data was analyzed using Skyline software (v 22.2) (7).

References

1. Saraswat M, Garapati K, Mun DG, Pandey A. Extensive heterogeneity of glycopeptides in plasma revealed by deep glycoproteomic analysis using size-exclusion chromatography. Mol Omics 2021;17:6:939-47.
2. Yang W, Shah P, Hu Y, Toghi Eshghi S, Sun S, Liu Y, Zhang H. Comparison of Enrichment Methods for Intact N- and O-Linked Glycopeptides Using Strong Anion

Exchange and Hydrophilic Interaction Liquid Chromatography. Anal Chem 2017;89:21:11193-7.

3. UniProt C. UniProt: the universal protein knowledgebase in 2021. Nucleic Acids Res 2021;49:D1:D480-D9.

4. Sugio S, Kashima A, Mochizuki S, Noda M, Kobayashi K. Crystal structure of human serum albumin at 2.5 A resolution. Protein Eng 1999;12:6:439-46.

5. Berman HM, Battistuz T, Bhat TN, Bluhm WF, Bourne PE, Burkhardt K, et al. The Protein Data Bank. Acta Crystallogr D Biol Crystallogr 2002;58:Pt 6 No 1:899-907.

6. Schrodinger, LLC. The PyMOL Molecular Graphics System, Version 1.8. 2015.

7. Pino LK, Searle BC, Bollinger JG, Nunn B, MacLean B, MacCoss MJ. The Skyline ecosystem: Informatics for quantitative mass spectrometry proteomics. Mass Spectrom Rev 2020;39:3:229-44.
