# Supplementary material for: Defining albumin as a glycoprotein with multiple N-linked glycosylation sites
Source: J Transl Med. 2024 May 13;22:454. doi: 10.1186/s12967-024-05000-5 (PMC11090807; doi:10.1186/s12967-024-05000-5)
Supplement: Supplementary file 2 — Additional file 2: Additional figures. Additional supporting figures providing additional information on the glycopeptides identified by discovery analysis of human serum, bovine serum albumin and rabbit serum albumin samples. [file 12967_2024_5000_MOESM2_ESM.pdf]

Additional File 2

Defining albumin as a glycoprotein with multiple N-linked glycosylation sites

Kishore Garapati[1,2,3,*], Anu Jain[3,*], Benjamin J. Madden[4], Dong-Gi Mun[3], Jyoti Sharma[1,2], Rohit Budhraja[3], Akhilesh Pandey[3,5,§]

[1]Manipal Academy of Higher Education (MAHE), Manipal, Karnataka, India
[2]Institute of Bioinformatics, International Technology Park, Bangalore, Karnataka, India

[3]Department of Laboratory Medicine and Pathology, Mayo Clinic, Rochester, Minnesota, USA
[4]Proteomics Core, Mayo Clinic, Rochester, Minnesota, USA
[5]Center for Individualized Medicine, Mayo Clinic, Rochester, Minnesota, United States

*These authors contributed equally

§Corresponding Author

Akhilesh Pandey, M.D., Ph.D.
Department of Laboratory Medicine and Pathology
Mayo Clinic
200 First Street SW
Rochester, MN 55905, USA
Tel: +1-507-293-9564
Email: pandey.akhilesh@mayo.edu

Figure S1

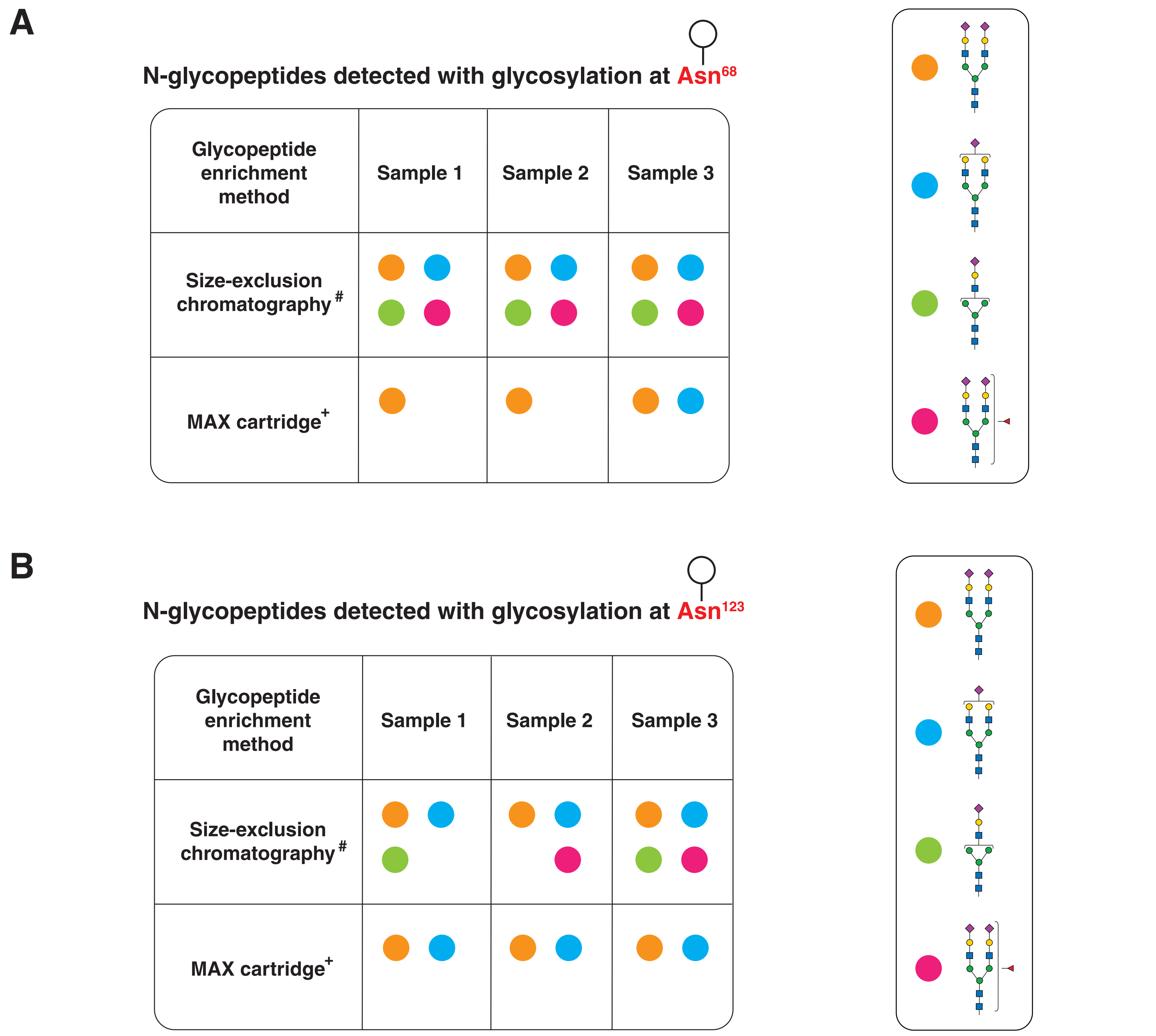

Glycopeptide enrichment from 4 mg serum protein digest each; 9 fractions analyzed by LC-MS/MS
+ Glycopeptide enrichment from 600 ug serum protein digest each; 1 fraction analyzed by LC-MS/MS

Glycans detected at each site by different analytical workflows. A. Glycopeptides with glycosylation at Asn68 were detected in the three analyzed samples with the glycans represented as shown, by SEC- and MAX-based glycopeptide enrichment methods. B. Glycopeptides with glycosylation at Asn123 were detected in the three analyzed samples with the glycans represented as shown, by SEC- and MAX-based glycopeptide enrichment methods.

Figure S2A

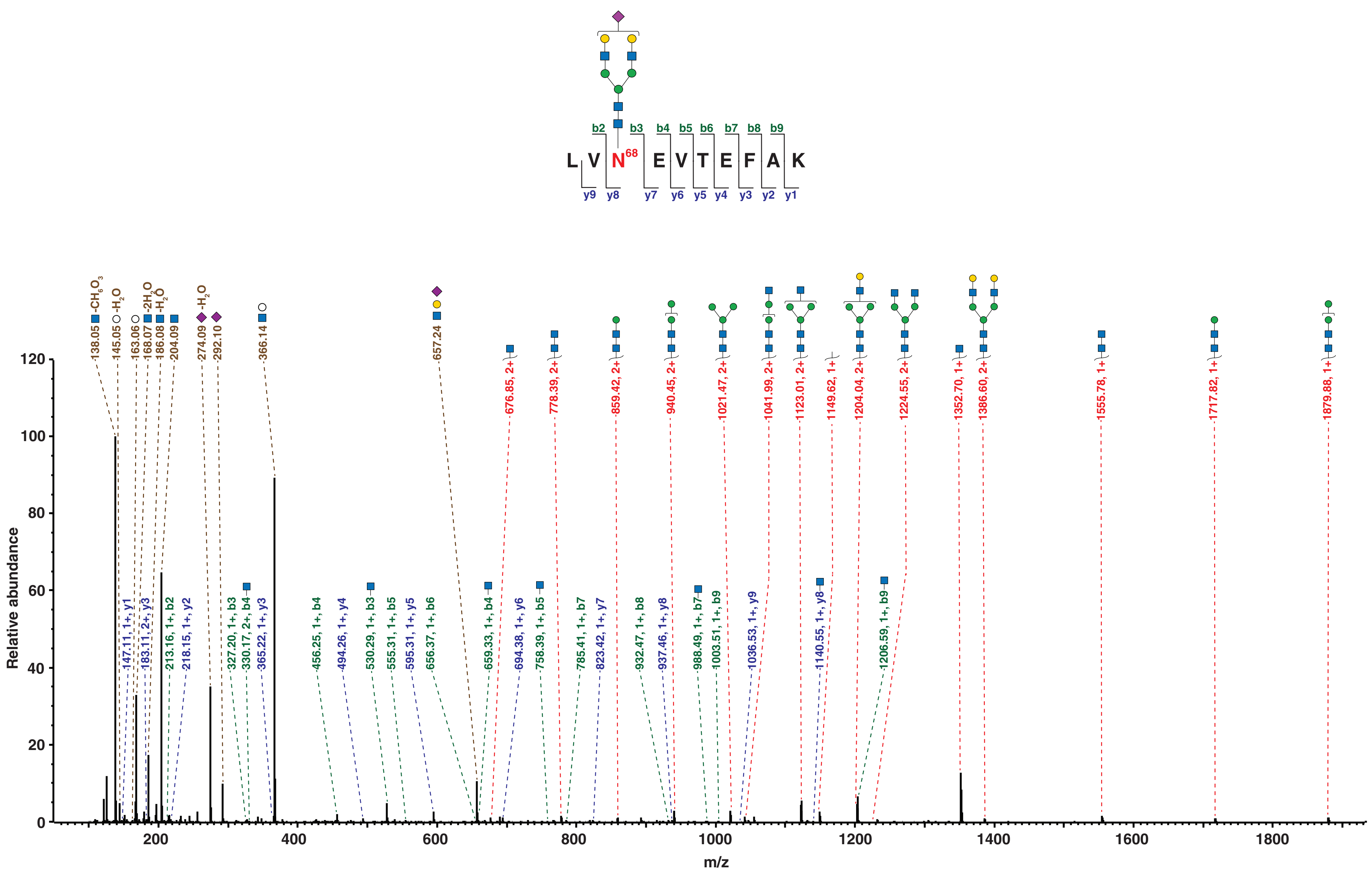

Annotated MS/MS spectrum of human albumin-derived glycopeptide with glycan Hex5HexNAc4NeuAc1 at Asn68

Figure S2B

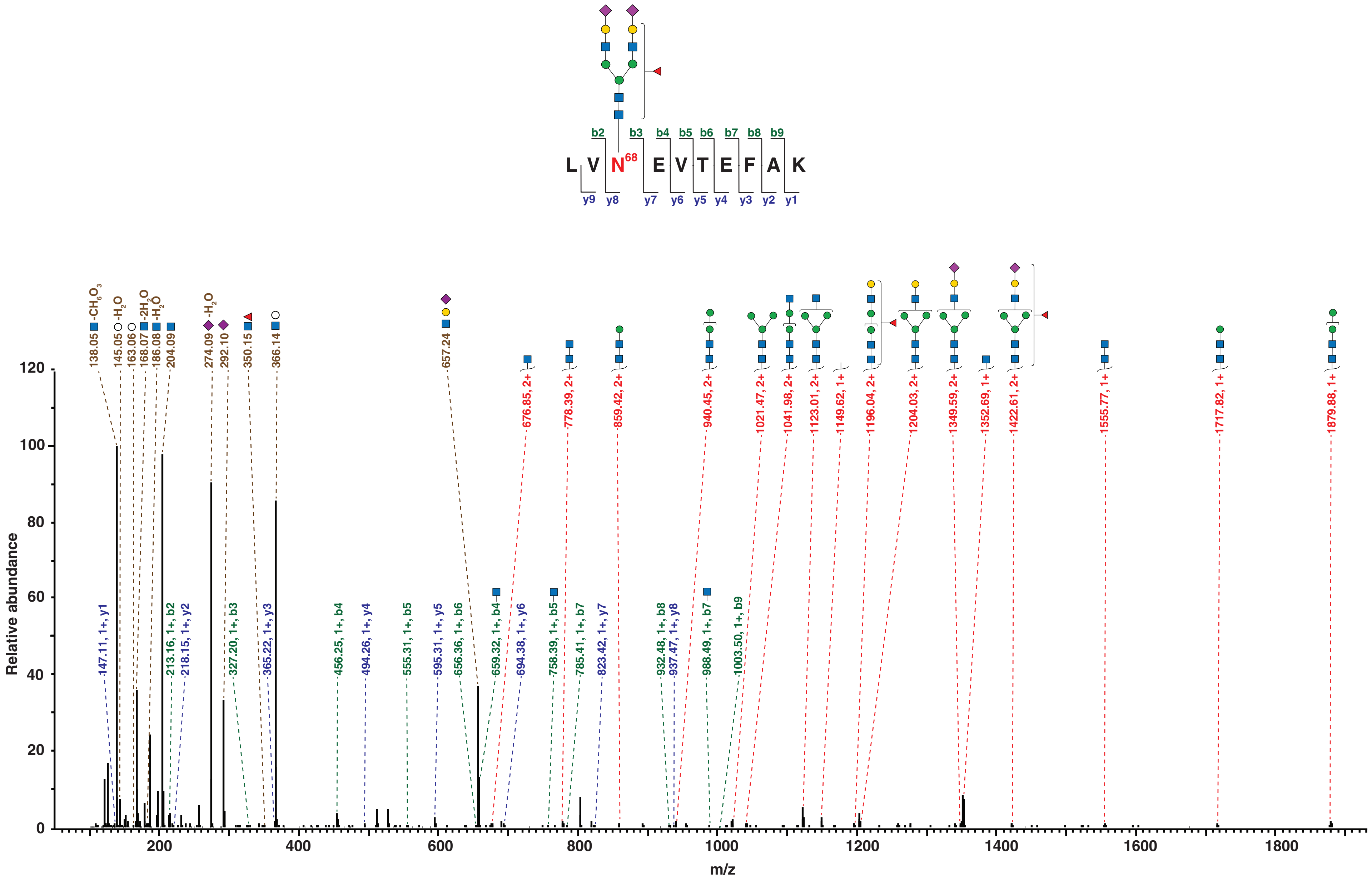

Annotated MS/MS spectrum of human albumin-derived glycopeptide with glycan Hex5HexNAc4NeuAc2Fuc1 at Asn68

Figure S2C

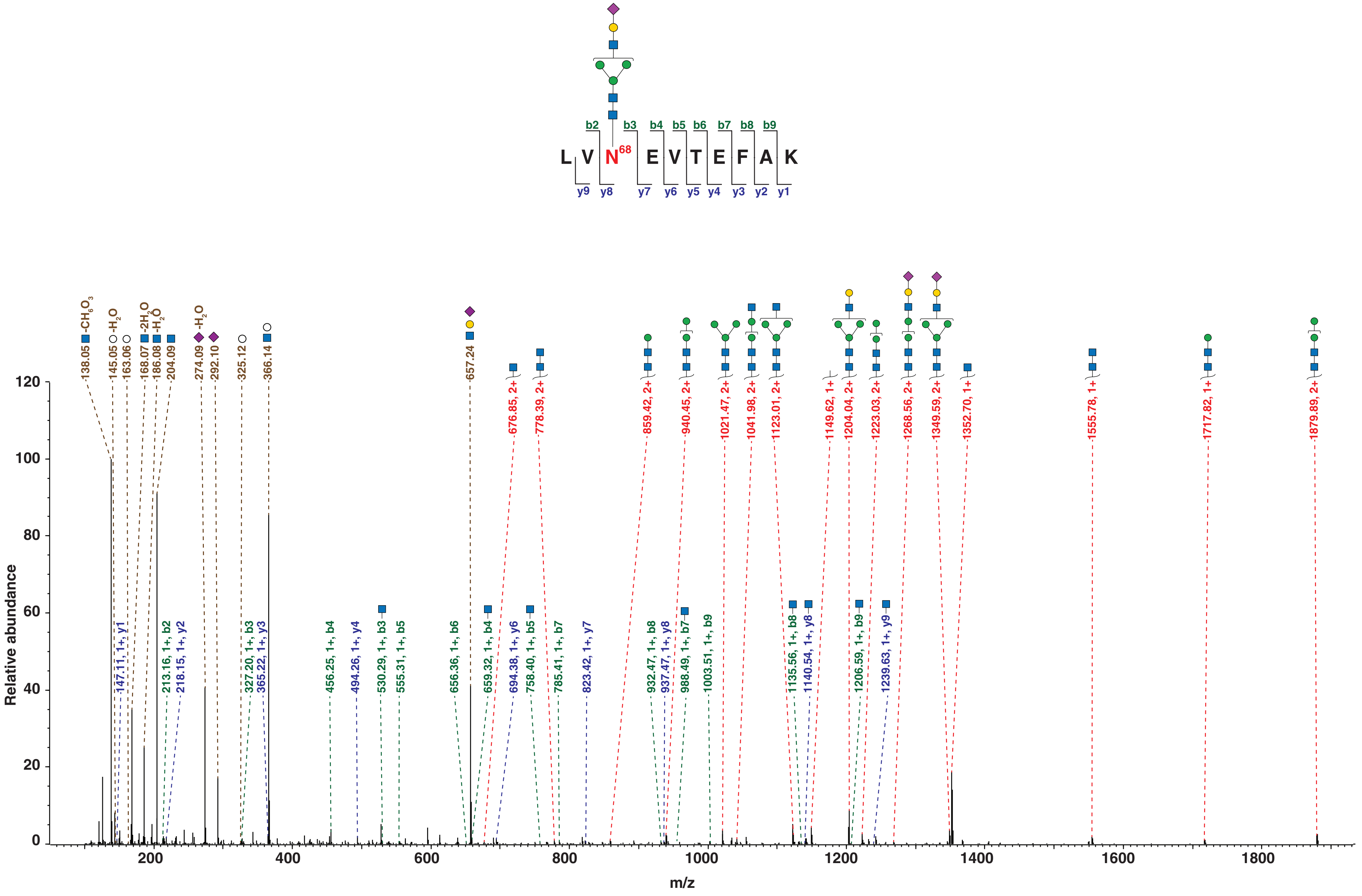

Annotated MS/MS spectrum of human albumin-derived glycopeptide with glycan Hex4HexNAc3NeuAc1 at Asn68

Figure S2D

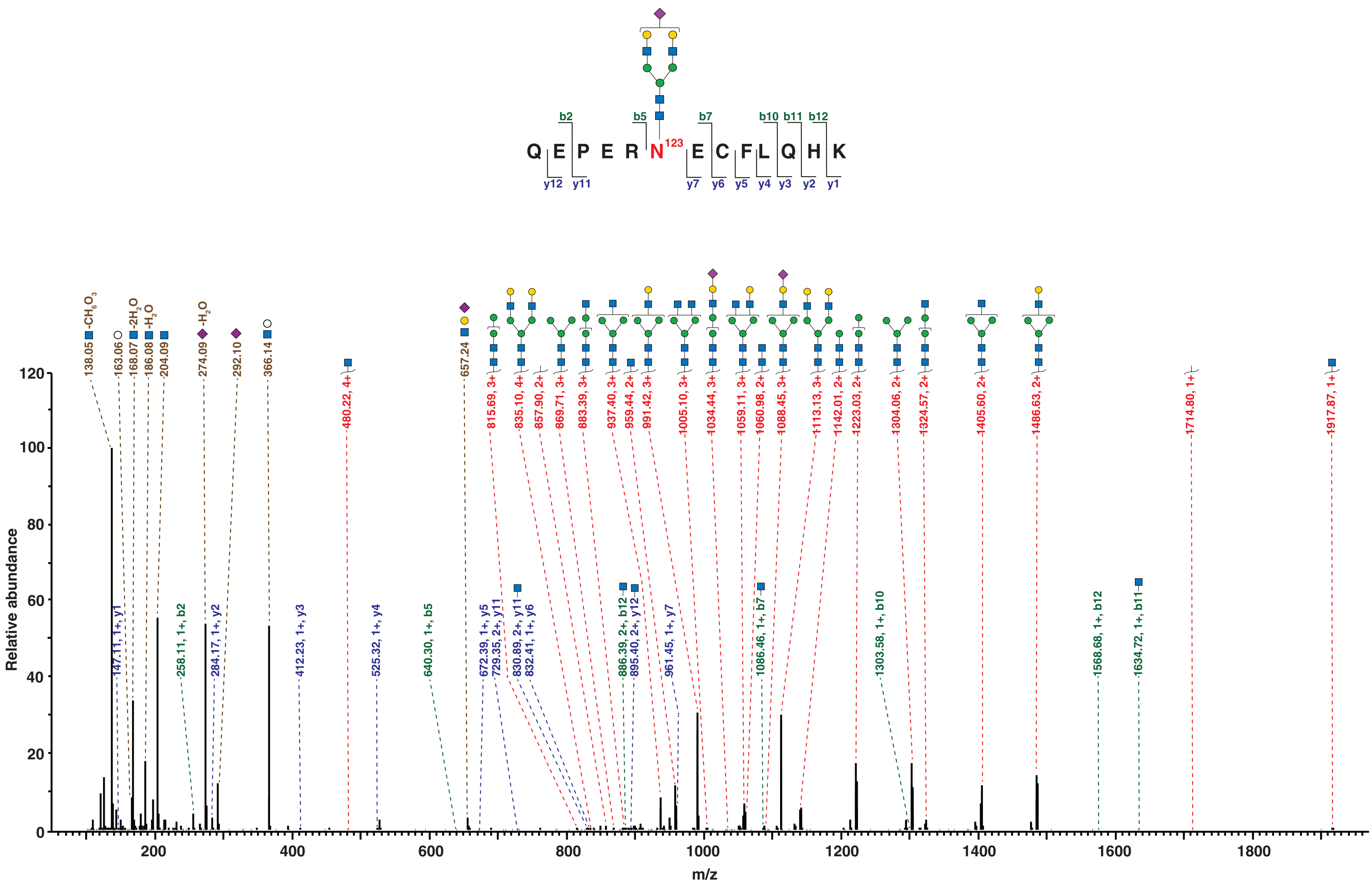

Annotated MS/MS spectrum of human albumin-derived glycopeptide with glycan Hex5HexNAc4NeuAc1 at Asn123

Figure S2E

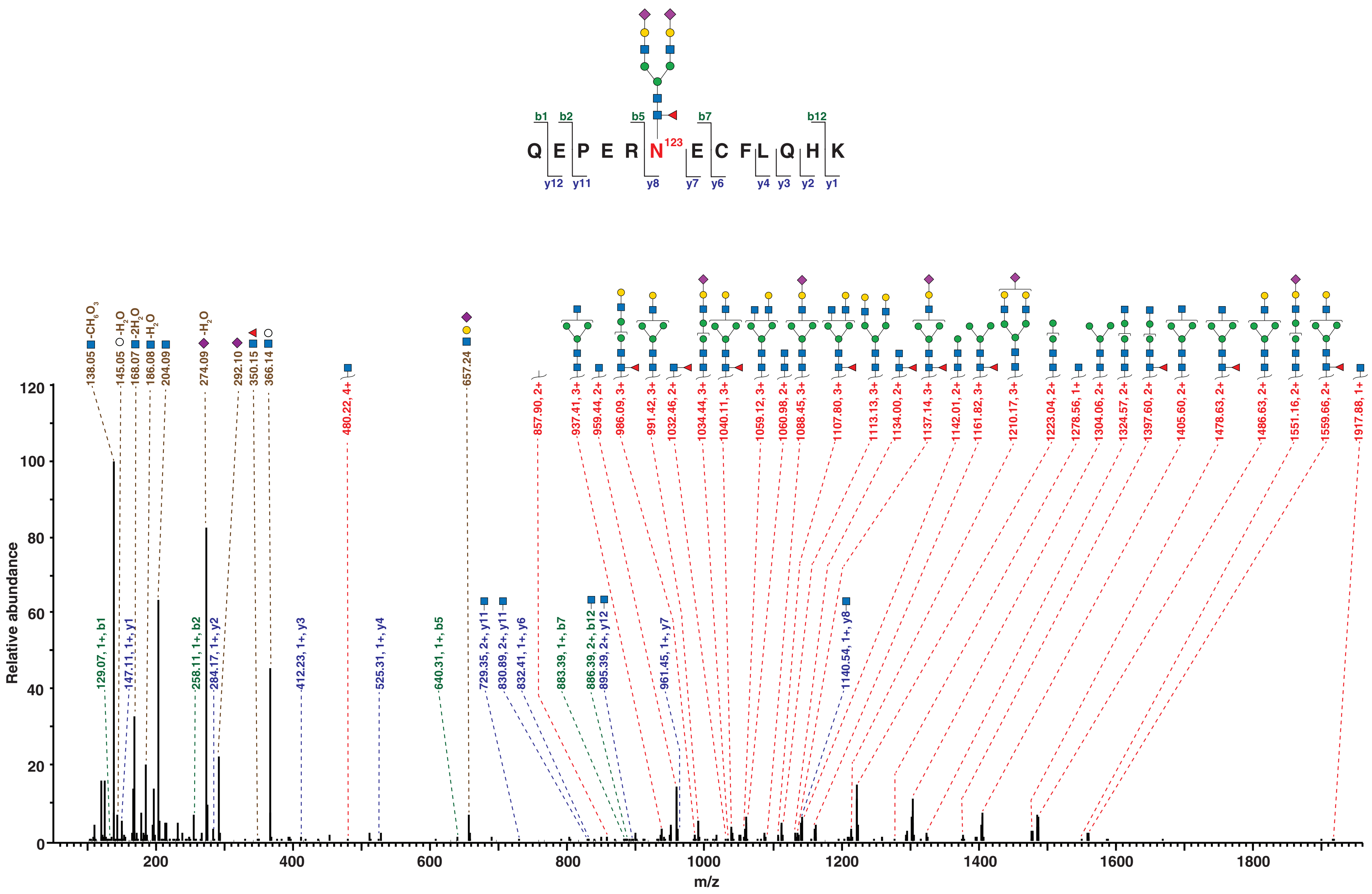

Annotated MS/MS spectrum of human albumin-derived glycopeptide with glycan Hex5HexNAc4NeuAc2Fuc1 at Asn123

Figure S2F

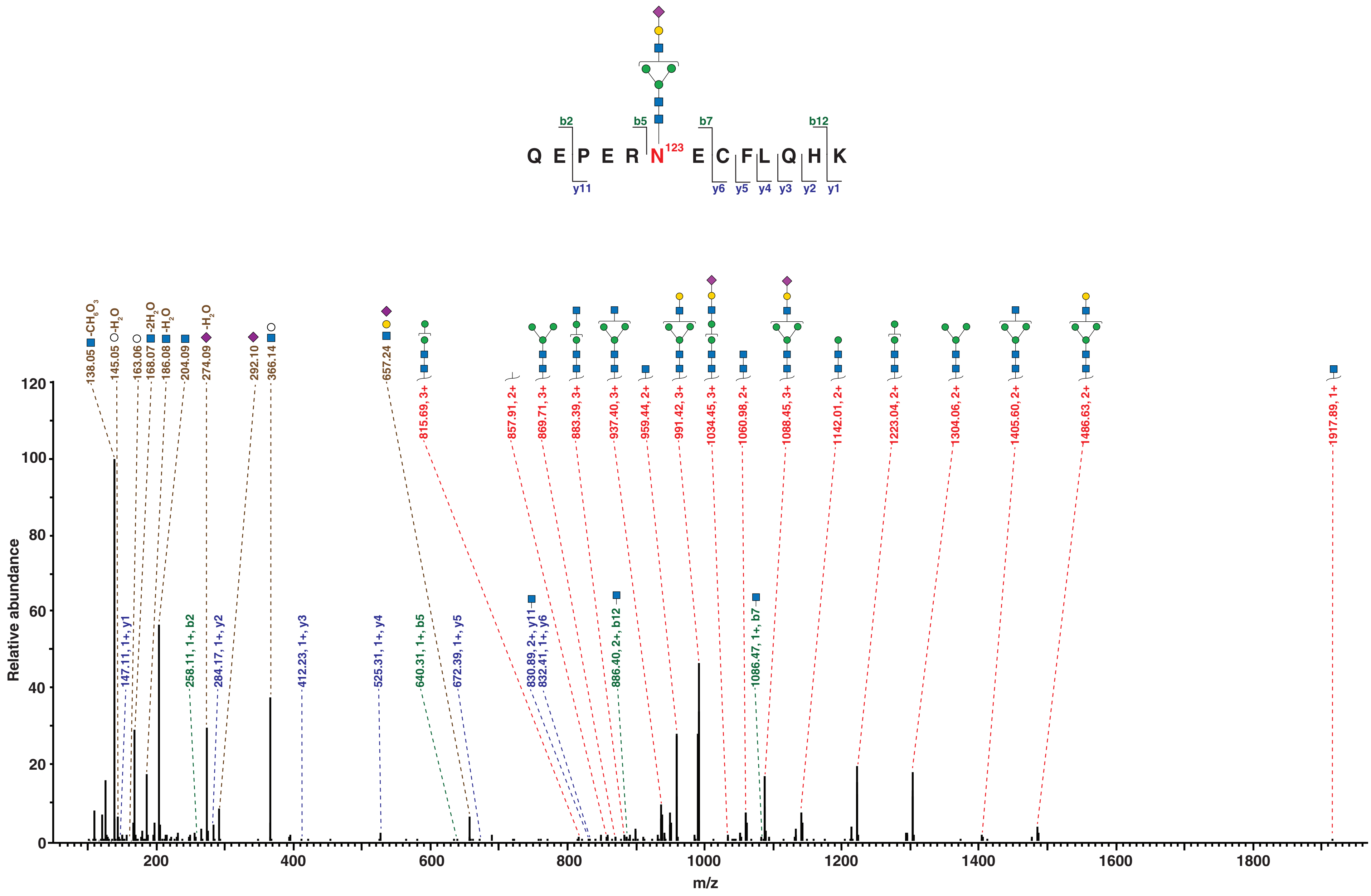

Annotated MS/MS spectrum of human albumin-derived glycopeptide with glycan Hex4HexNAc3NeuAc1 at Asn123

Figure S3A

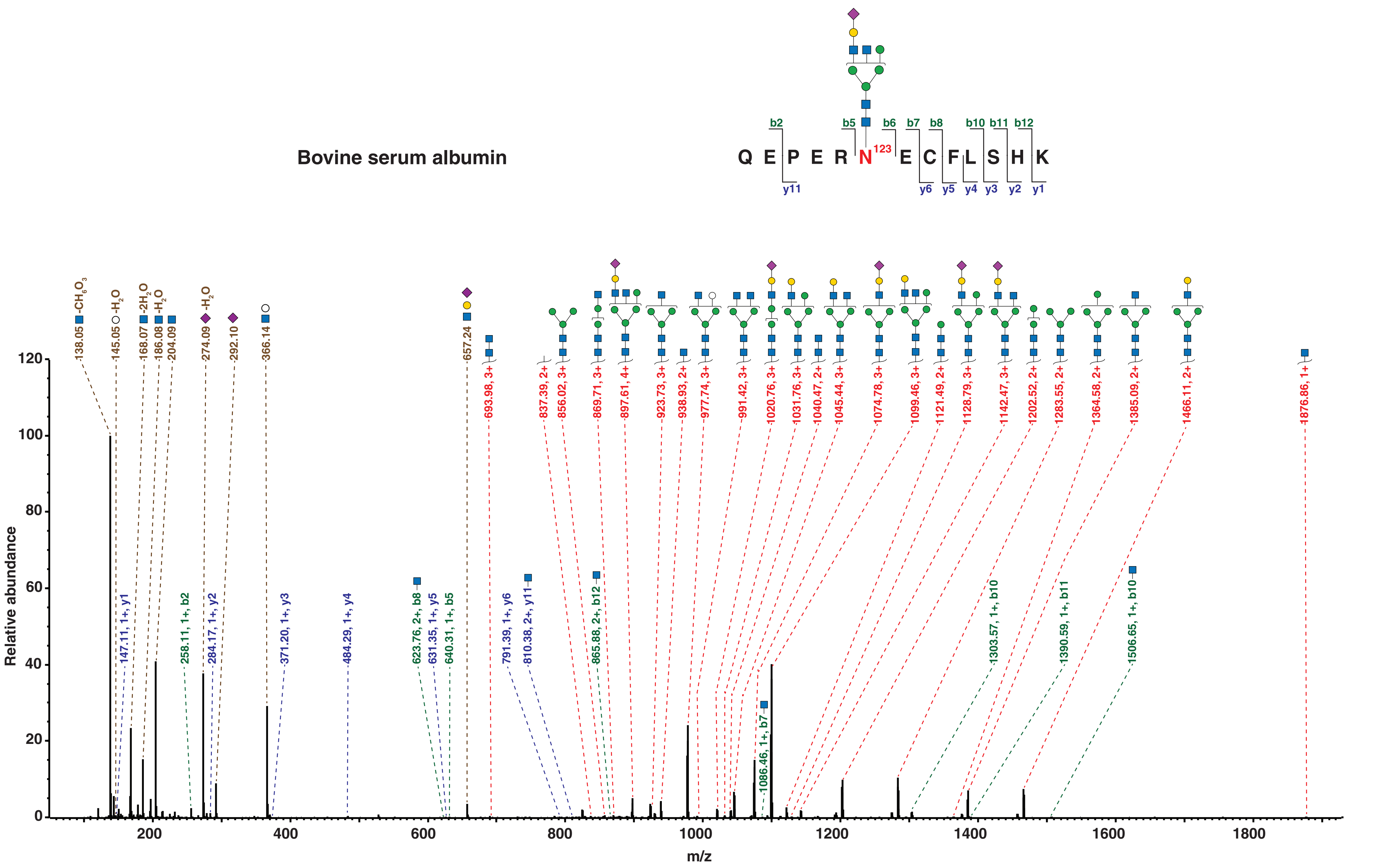

Annotated MS/MS spectrum of bovine serum albumin-derived glycopeptide with glycan Hex5HexNAc4NeuAc1 at Asn123

Figure S3B

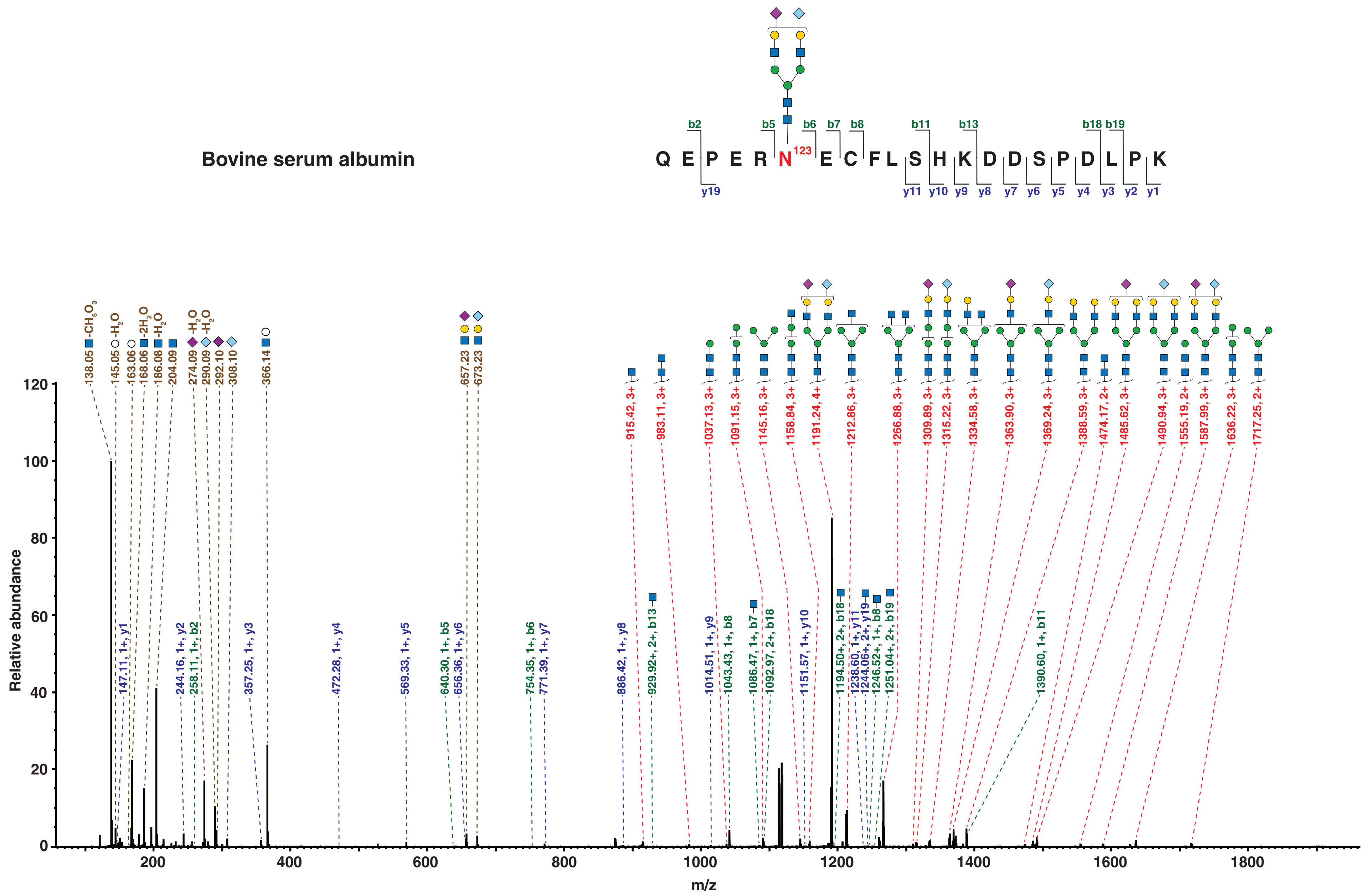

Annotated MS/MS spectrum of bovine serum albumin-derived glycopeptide with glycan Hex5HexNAc4NeuAc1NeuGc1 at Asn123

Figure S3C

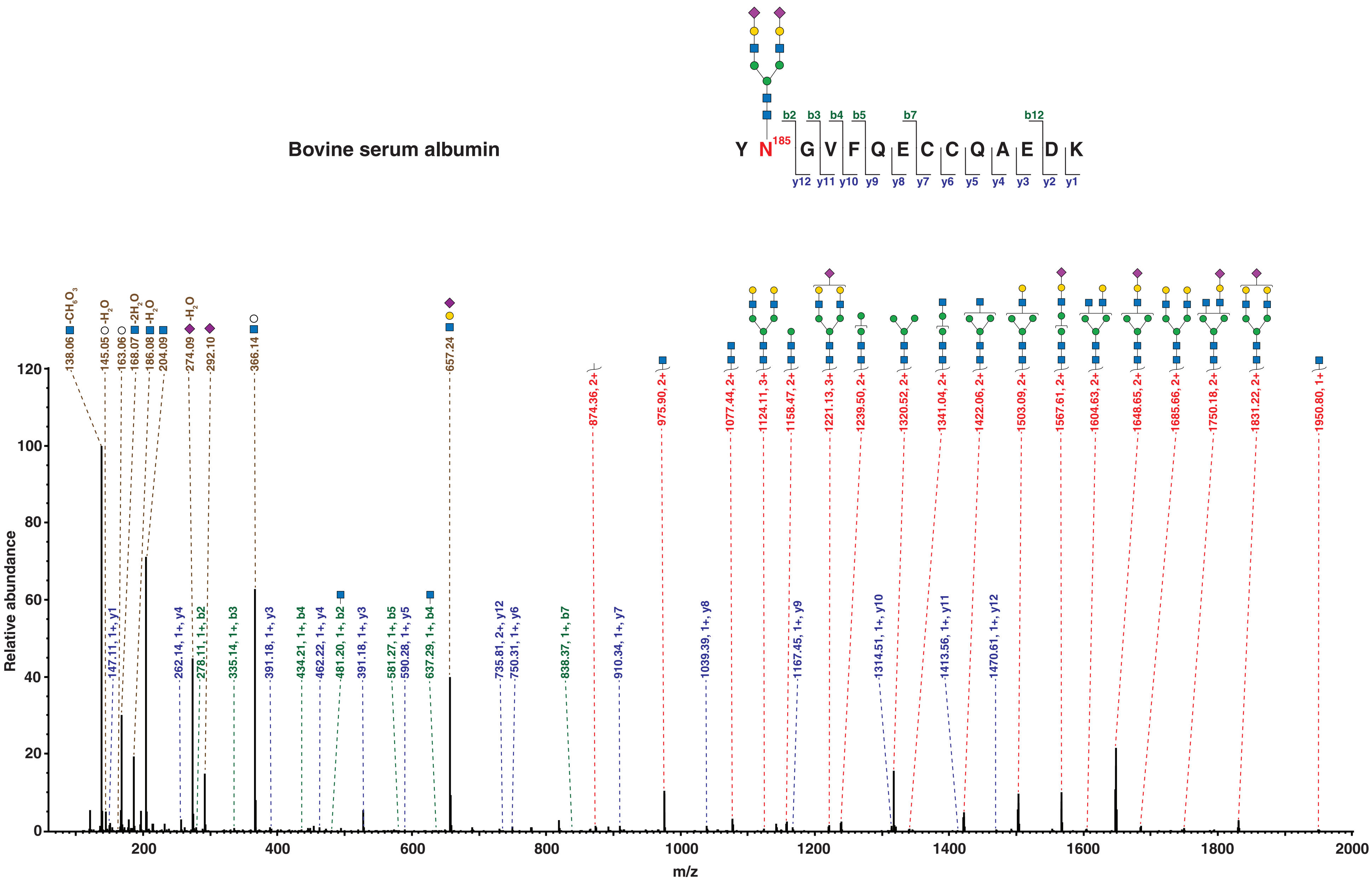

Annotated MS/MS spectrum of bovine serum albumin-derived glycopeptide with glycan Hex5HexNAc4NeuAc2 at Asn185

Figure S3D

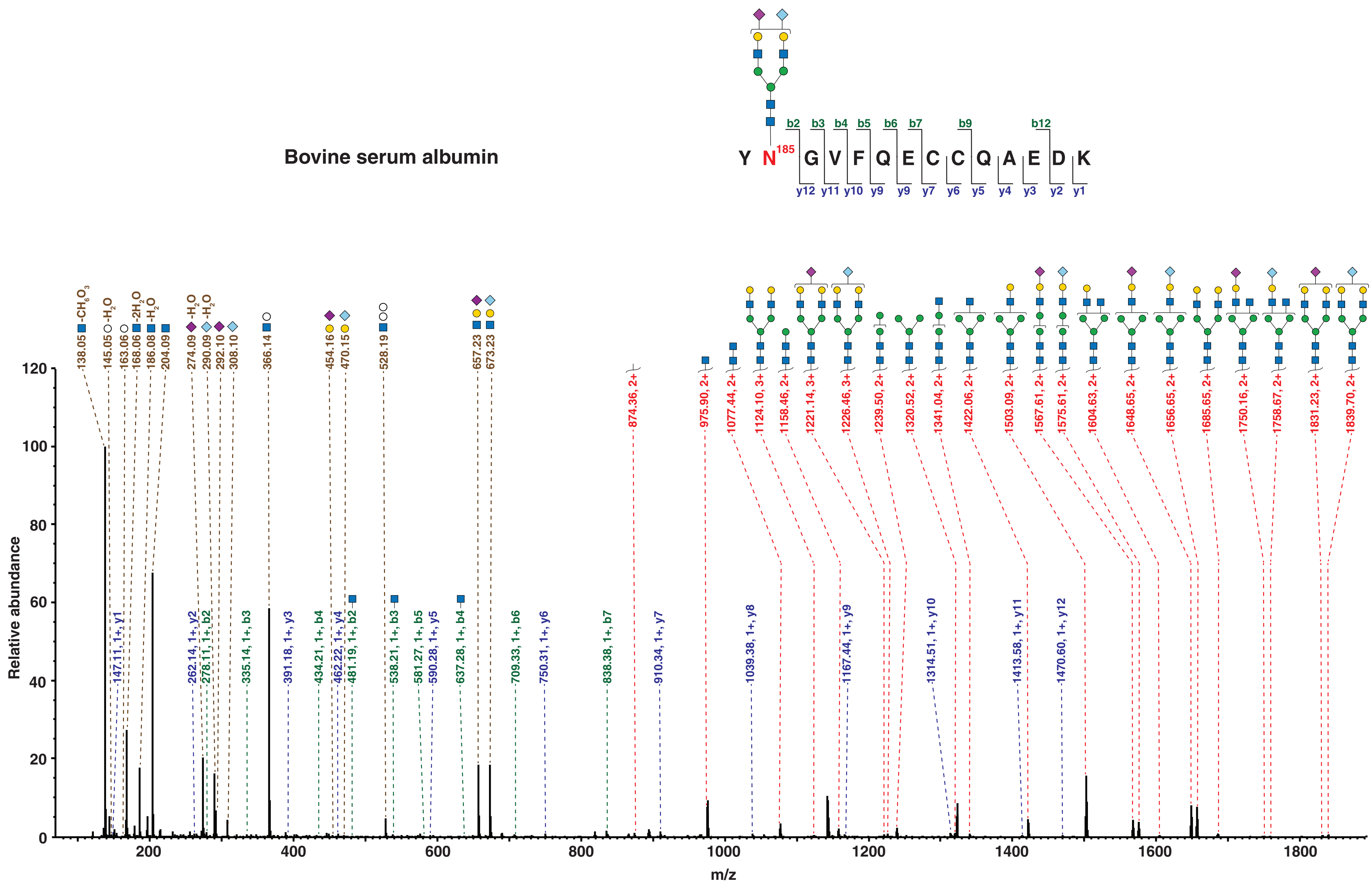

Annotated MS/MS spectrum of bovine serum albumin-derived glycopeptide with glycan Hex5HexNAc4NeuAc1NeuGc1 at Asn185

Figure S3E

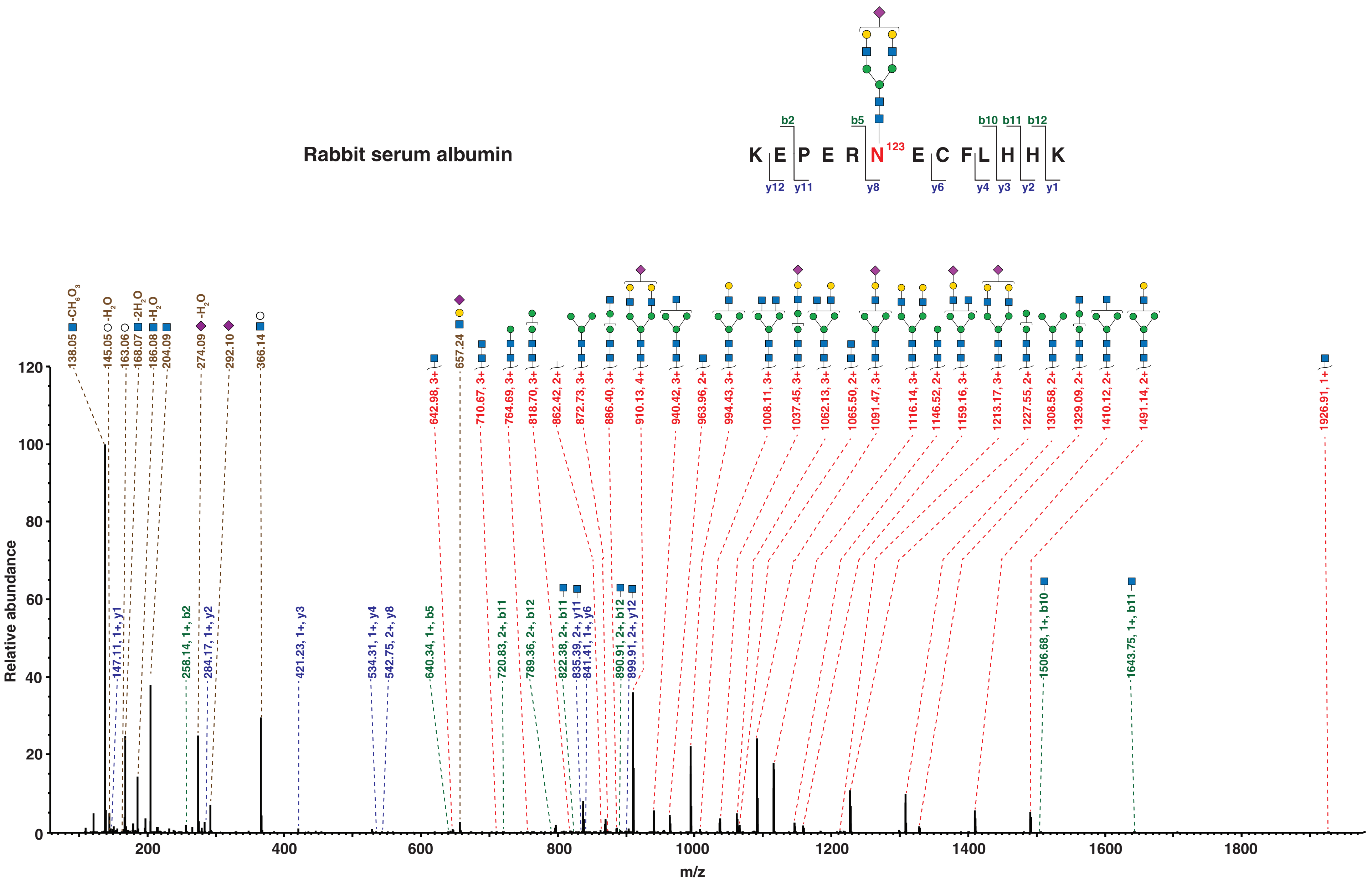

Annotated MS/MS spectrum of rabbit serum albumin-derived glycopeptide with glycan Hex5HexNAc4NeuAc1 at Asn123
